# Functional near-infrared spectroscopy for the assessment and treatment of patients with disorders of consciousness

**DOI:** 10.3389/fneur.2025.1524806

**Published:** 2025-02-03

**Authors:** Nan Wang, Yifang He, Sipeng Zhu, Dongsheng Liu, Xiaoke Chai, Qiheng He, Tianqing Cao, Jianghong He, Jingqi Li, Juanning Si, Yi Yang, Jizong Zhao

**Affiliations:** ^1^Department of Neurosurgery, Beijing Tiantan Hospital, Capital Medical University, Beijing, China; ^2^Department of Neurosurgery, Peking Union Medical College Hospital, Chinese Academy of Medical Sciences and Peking Union Medical College, Beijing, China; ^3^China National Clinical Research Center for Neurological Diseases, Beijing, China; ^4^School of Instrumentation Science and Opto-Electronics Engineering, Beijing Information Science and Technology University, Beijing, China; ^5^Clinical College of Neurology, Neurosurgery and Neurorehabilitation, Tianjin Medical University, Tianjin, China; ^6^Brain Computer Interface Transitional Research Center, Beijing Tiantan Hospital, Capital Medical University, Beijing, China; ^7^Hangzhou Mingzhou Brain Rehabilitation Hospital, Hangzhou, China; ^8^China National Center for Neurological Disorders, Beijing, China; ^9^National Research Center for Rehabilitation Technical Aids, Beijing, China; ^10^Chinese Institute for Brain Research, Beijing, China; ^11^Beijing Institute of Brain Disorders, Beijing, China

**Keywords:** functional near-infrared spectroscopy, disorders of consciousness, neuromodulation, brain-computer interface, monitoring and assessment

## Abstract

**Background:**

Advances in neuroimaging have significantly enhanced our understanding of brain function, providing critical insights into the diagnosis and management of disorders of consciousness (DoC). Functional near-infrared spectroscopy (fNIRS), with its real-time, portable, and noninvasive imaging capabilities, has emerged as a promising tool for evaluating functional brain activity and nonrecovery potential in DoC patients. This review explores the current applications of fNIRS in DoC research, identifies its limitations, and proposes future directions to optimize its clinical utility.

**Aim:**

This review examines the clinical application of fNIRS in monitoring DoC. Specifically, it investigates the potential value of combining fNIRS with brain-computer interfaces (BCIs) and closed-loop neuromodulation systems for patients with DoC, aiming to elucidate mechanisms that promote neurological recovery.

**Methods:**

A systematic analysis was conducted on 155 studies published between January 1993 and October 2024, retrieved from the Web of Science Core Collection database.

**Results:**

Analysis of 21 eligible studies on neurological diseases involving 262 DoC patients revealed significant findings. The prefrontal cortex was the most frequently targeted brain region. fNIRS has proven crucial in assessing brain functional connectivity and activation, facilitating the diagnosis of DoC. Furthermore, fNIRS plays a pivotal role in diagnosis and treatment through its application in neuromodulation techniques such as deep brain stimulation (DBS) and spinal cord stimulation (SCS).

**Conclusion:**

As a noninvasive, portable, and real-time neuroimaging tool, fNIRS holds significant promise for advancing the assessment and treatment of DoC. Despite limitations such as low spatial resolution and the need for standardized protocols, fNIRS has demonstrated its utility in evaluating residual brain activity, detecting covert consciousness, and monitoring therapeutic interventions. In addition to assessing consciousness levels, fNIRS offers unique advantages in tracking hemodynamic changes associated with neuroregulatory treatments, including DBS and SCS. By providing real-time feedback on cortical activation, fNIRS facilitates optimizing therapeutic strategies and supports individualized treatment planning. Continued research addressing its technical and methodological challenges will further establish fNIRS as an indispensable tool in the diagnosis, prognosis, and treatment monitoring of DoC patients.

## 1 Introduction

Although clinical advancements have significantly reduced mortality rates, they have also led to an increase in the number of patients with disorders of consciousness (DoC) (1). Patients with DoC require prolonged and intensive clinical management, imposing a significant burden on families and society. DoC encompasses conditions characterized by diminished awareness or impaired cognition of oneself and the surrounding environment, including coma, vegetative state (VS)/ unresponsive wakefulness syndrome (UWS), and minimally conscious state (MCS). Patients in a VS/UWS exhibit intermittent wakefulness without any signs of self-awareness or environmental recognition (2). In contrast, patients in an MCS demonstrate fluctuating but reproducible signs of conscious awareness through clinical motor responses, although their level of awareness is unstable (3). MCS is further subdivided into MCS+ and MCS−, reflecting higher and lower levels of behavioral responsiveness, respectively (4, 5). The Coma Recovery Scale-Revised (CRS-R) is widely regarded as the most reliable clinical tool for diagnosing patients with prolonged DoC (6). However, clinical consensus-based assessments are associated with a misdiagnosis rate of approximately 40% (7).

Patients with DoC require prolonged and intensive clinical management, imposing a significant burden on families and society. In the past few years, the advancement of neuroimaging techniques such as functional magnetic resonance imaging (fMRI), positron emission tomography (PET), and electroencephalography (EEG) has provided objective diagnostic tools for assessing cognitive function, predicting neurological recovery, and determining prognosis in patients with disorders of consciousness (8, 9). EEG offers advantages in its superior temporal resolution and real-time responsiveness, however, its low spatial resolution and susceptibility to artifacts limit the diagnostic accuracy of DoC. In contrast, fMRI and PET provide high spatial resolution but are hindered by high costs, low portability, and limited real-time responsiveness (10, 11). Functional near-infrared spectroscopy (fNIRS) emerges as a promising alternative technique, offering a non-invasive, portable, and high temporal resolution for monitoring brain activity. Unlike fMRI and PET, fNIRS enables long-term bedside monitoring, making it particularly suitable for DoC patients. Additionally, fNIRS is less sensitive to motion artifacts than fMRI, enhancing its feasibility in clinical settings (12, 13). By facilitating early and accurate diagnosis, fNIRS provide valuable insights in guiding targeted interventions, reducing unnecessary treatments, shortening hospitalization durations, and alleviating the financial and logistical burden on healthcare systems. Ultimately, it optimizes healthcare resource allocation for DoC patients.

fNIRS is a non-invasive optical neuroimaging technique that utilizes near-infrared light (700–900 nm) to measure changes in tissue concentrations of oxy-hemoglobin (HbO), deoxy-hemoglobin (HbR), and total-oxyhemoglobin (HbT), providing (14–16). The selection of near-infrared light is based on its unique optical properties rather than its penetration depth. Within the near-infrared spectrum, light exhibits minimal absorption by water and significant absorption differences between HbO and HbR, enabling fNIRS to differentiate chromophores effectively. This “optical window” allows light to penetrate several centimeters into cortical tissue, facilitating reliable measurements of hemodynamic changes in highly scattering biological tissues (17–19). Advancements in fNIRS instrumentation, including continuous wave, frequency domain, and time domain systems, have further improved its sensitivity and broadened its clinical and research applications. These advancements have enabled the integration of fNIRS with other modalities, such as EEG, for comprehensive assessments of brain function (20, 21) fNIRS offers several strengths, including its non-invasive nature, portability, real-time measurement capabilities, and continuous repeatability (22–24). Compared to fMRI, fNIRS is less sensitive to interference from devices such as hearing aids, pacemakers, or cochlear implants, making it valuable for monitoring scalp oxygenation in patients with such medical implants (25). fNIRS has demonstrated its utility in identifying functional differences in key brain regions, including the occipital lobe, temporal lobe, and prefrontal lobe, in patients with various types of DoCs (26, 27). Moreover, it can detect neural responses to active stimuli in MCS and assess brain activity during verbal and motor imagery (MI) tasks, providing critical insights into residual cognitive and functional capacities (28–30).

**Figure 1:**
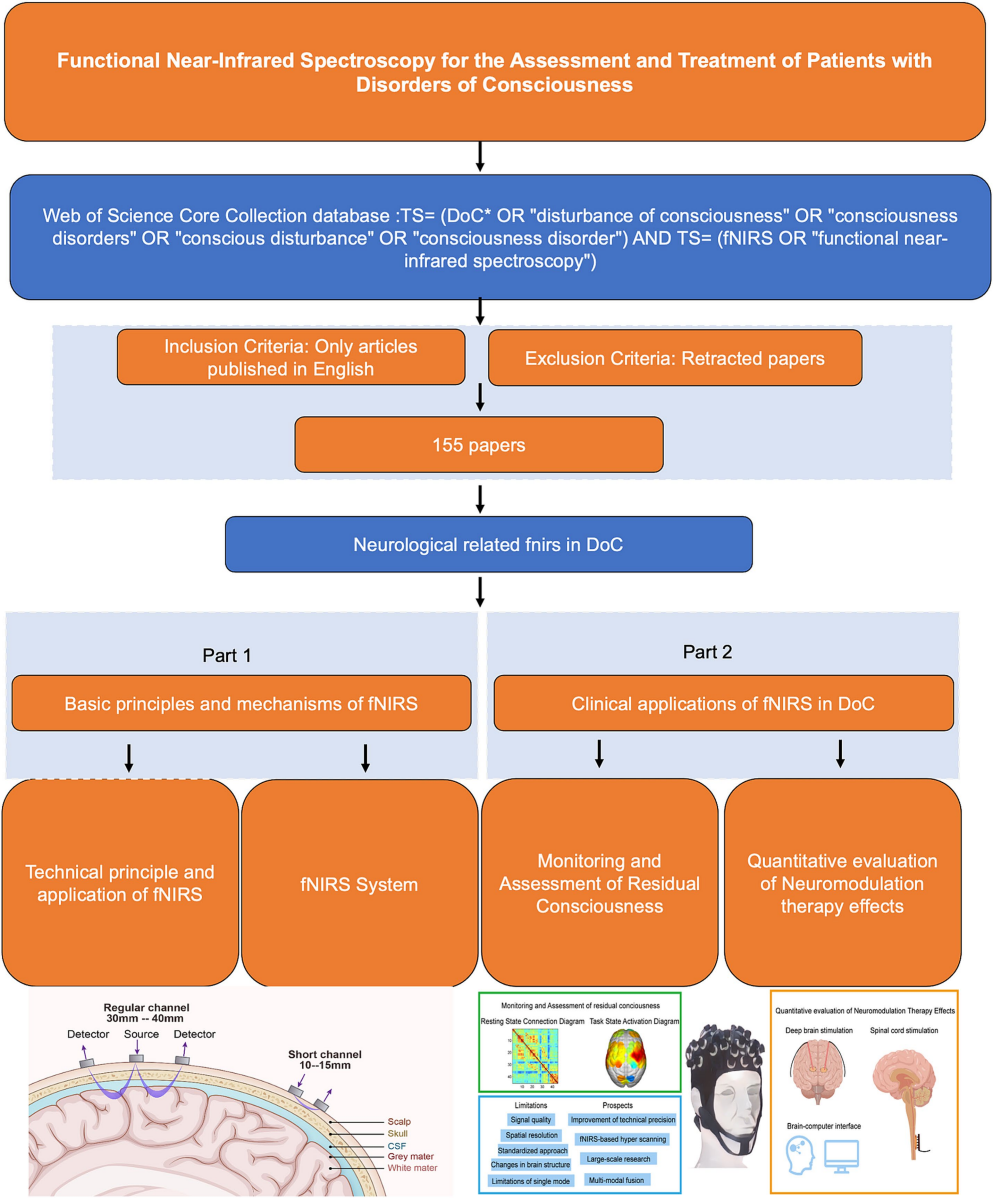
Flow chart.

**Figure 2:**
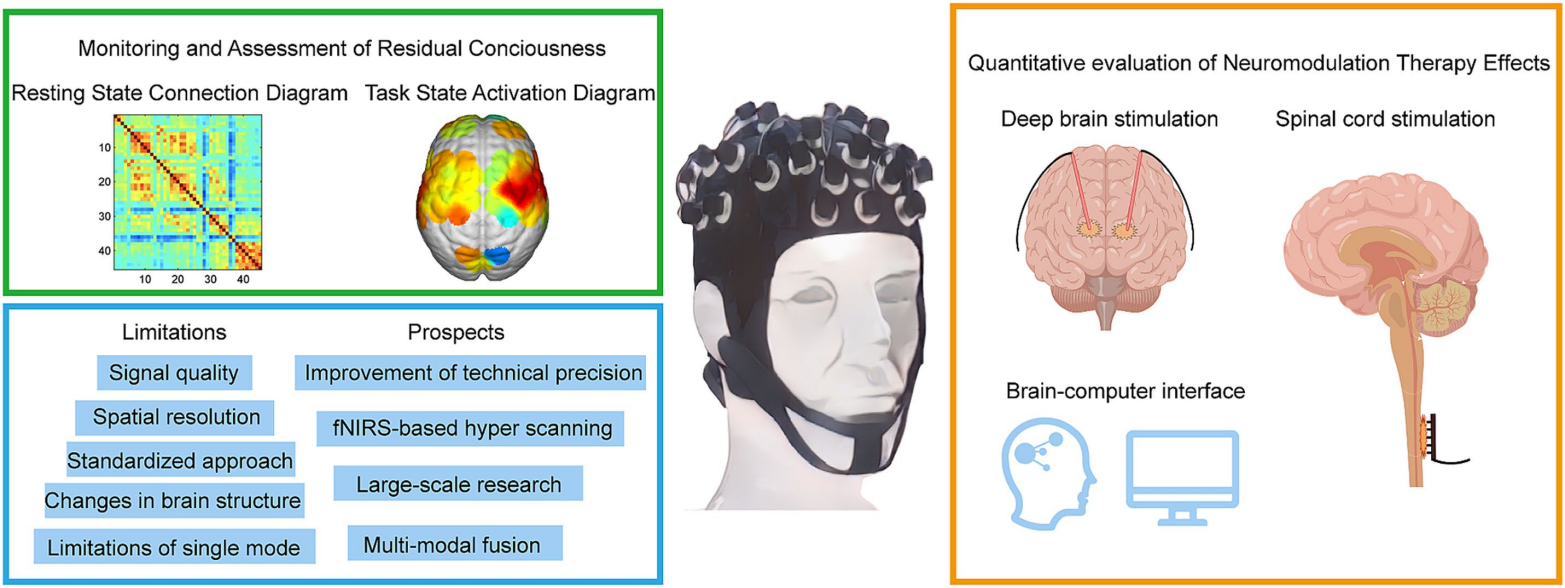
Methods for diagnosing and assessing different levels of DoC by fNIRS. fNIRS technology has made recent advances in the direction of deep brain electrical stimulation, spinal cord electrical stimulation, and brain-computer interfaces. fNIRS has problems and challenges, as well as trends in future clinical applications.

As a non-invasive neuroimaging technique, fNIRS has gained significant attention in the field of DoC. It has demonstrated high sensitivity in diagnosing varying degrees of DoC, evaluating and predicting changes in brain function, and monitoring therapeutic outcomes (31). Notably, fNIRS offers unique advantages in assessing the effects of neuromodulator therapies on DoC patients (32, 33). By providing real-time insights into neural responses and treatment efficacy, fNIRS serves as an essential tool for guiding and optimizing treatment strategies. This review provides an overview of the clinical applications of fNIRS from 1993 to 2024, with a focus on the latest advances in fNIRS technology for DoC patient assessment, functional neuromodulation therapies, and brain-computer interfaces (BCIs) (Table 1). The flowchart in Figure 1 illustrates the search strategy, including keyword retrieval and paper screening processes. Additionally, recent clinical studies are summarized in Table 2, with data sourced from the World Health Organization (WHO) International Clinical Trials Registry Platform (ICTRP^1^). This review also explores emerging trends in clinical applications while addressing the current challenges and limitations of using fNIRS to inform DoC treatment strategies (Figure 2).

**Table 1:** Summary of fNIRS studies conducted on DoC patients to date.

Title	General characteristics of the patients							fNIRS			
	Sample sex/age	Sample size (VS/MCS)	Etiology	Duration of DoC (months)	Types of external stimuli	Task type	Number of repetitions or measurement duration	fNIRS system (CW/ wavelengths/sample rate)	Brain regions of interest for probe emission	Channels rate of SD	Type of analysis
Zhao et al. (65)	15 DoC:7 F and 8 M; 16 HC	15 MCS	5 TBI, 10 Intracerebral infarction	1 ~ 12 months	Resting state	Passive paradigms	5 min	CW, 690 and 830 nm, 20 Hz	Both sides of PreM and SMA, FEF, Broca’s area, FPA, and DLPFC	53 channels	Network analysis
Liu et al. (80)	23 DoC:8 F and 15 M,50 ± 19 years 24 HC:8 F and 16 M,30 ± 7 years	11 UWS/VS and 12 MCS	4 TBI, 5 Encephalitis, 6 HIE, 8 Vascular	27 ~ 138 days	Resting state		5 min	CW, 695 and 830 nm, 100 Hz	PFC was selected: both sides of BA 9, BA10, BA44, BA45, and BA46	28 channels	Network analysis
Moriya et al. (117)	8 DoC:6 F and 2 M,70.8 ± 11.8 years	8 DoC	8 Stroke	Within 2 weeks after stroke	Resting state		10 min	CW	The bilateral PFC	2 channels	Functional Connectivity Analysis
Bicciato et al. (118)	22 DoC:15 F and 7 M,68.8 ± 10.9 years 6 HC:2 F and 4 M,41.2 ± 12.6 years	5 VS/UWS and 17 MCS	8 aSAH, 2 ischemic stroke, 2 Encephalitis, 4 ICH, 4 NCSE, 1 SDH, 1 Encephalitis	/	Resting state and music		Each block (resting state, music, resting state) lasted from 4 to 7 min.	/,25 Hz	The prefrontal brain	2 channels	Frequency domain analysis
Luo et al. (119)	10 pDoC:7 F and 6 M,51.50 (27.50, 64.50) 13 HC:5 F and 5 M,44.00 (27.00, 54.00)	10 pDoC	/	/	Resting state		5 min	CW,760 and 850 11 Hz	DAN, DMN, FPN, SEN, VAN, VIS.	63 channels	Functional Connectivity Analysis
Xin et al. (120)	16 DoC:9 F and 7 M, aged 60.75 ± 19.74 years 6 HC:2 F and 4 M, aged 22–33 years	10 VS and 6 MCS	/	1 ~ 48 months	Acupuncture		3 blocks	CW,730 and 850 nm,28 Hz	PFC	48 channels	Network analysis
Liu et al. (121)	28 pDoC:18 M and 10 F	9 COMA, 7 VS and 12 MCS	7 TBI, 10 Hemorrhagic injury, 11 Ischemic injury	A:8.0 ~ 9.5 months B:7.1 ~ 82.2 months	Acupuncture		8 blocks	CW,780、805 and 830 nm,13.33 Hz	DLPFC, PMC, and S1	20 channels	Functional Connectivity Analysis
Straudi et al. (122)	10 MCS:3 F and 7 M, aged 35.5 ± 12.6 years	10 MCS	10 TBI	5.5 ± 5.4 years	tDCS		2 blocks	CW,760 and 850 nm,3.47 Hz	Frontal brain regions	24 channels	Time-domain analysis
Zhang et al. (33)	9 DoC:4 F and 5 M, 17–64 years	7 VS and 2 MCS	2 Head trauma, 3 Cerebral hemorrhage, 1 Stroke, 3 Hypoxic ischemic encephalopathy	3 ~ 28 months	SCS		4 blocks	CW,690 and 830 nm,100 HZ	PFC	8 channels	Time-domain analysis
Si et al. (92)	10 DoC:5 F and 5 M, aged 17–64 years	8 VS and 2 MCS	2 Head trauma, 4 Anoxic, 1 Stroke, 1 Cerebral trauma, 2 Cerebral hemorrhage	4 ~ 28 months	SCS		5 frequencies, 4 blocks per frequency	CW,690 and 830 nm,100 HZ	PFC	4 channels	Time-domain analysis
Zhu et al. (87)	1 DoC, M, 78 years old	1 DoC	TBI	5 months	SCS		7 times	CW,730 and 850 nm,11 Hz	The prefrontal lobe	16 channels	Functional Connectivity Analysis
Lu et al. (32)	13 DoC:4 F and 9 M, aged 18–75 years	9 VS and 2 MCS−	6TBI, 1 Hypothalamus Hemorrhage, 1 Basal Ganglia Hemorrhage, 1 Cerebellar Hemorrhage, 2 Brainstem Hemorrhage, 2 Brainstem Infarction	3 ~ 18 months	DBS		4 blocks	CW,730 and 850 nm,11 Hz	The frontal, parietal, and occipital lobes	34 channels	Network analysis
Shu et al. (123)	11 DoC:2 F and 8 M, 24 HC	9 UWS/VS and 2 MCS−	4 Trauma, 2 Brainstem infarction, 5 Cerebral hemorrhage	3 ~ 18 months	DBS		2 times, 4 blocks each time	CW,730 and 850 nm,11 Hz	The frontal, parietal, and occipital lobes	34 channels	Network analysis
Shu et al. (91)	10 DoC:3 F and 7 M,37 ~ 75 years	8 VS and 2 MCS	4 Trauma, 4 Cerebral hemorrhage, 2 Brainstem infarction	3 ~ 18 months	DBS		2 times, 4 blocks each time	CW,760 and 850 nm,11 Hz	The frontal, parietal, and occipital lobes	34 channels	Network analysis
Yin et al. (124)	26 DoC:4 F and 22 M,27 ~ 99 years; 20 HC	26 MCS	1 HCP, 12 HIE, 5 CH, 4 MG, 4 CI	/	LF music (31–180 Hz) intervention, MF music (180–4 k Hz) intervention		3 blocks	CW,760 and 850 nm,10 Hz	DLPFC, PFC, PMC, S1, and M1	42 channels	Functional Connectivity Analysis
Lu et al. (125)	18 DoC:4 F and 14 M, 47.83 ± 12.88 years; 15 HC:8 F and 7 M, 25.93 ± 6.62 years	14 VS and 4 MC	8 Stroke, 5 TBI, 5 Anoxic	1.7 ~ 25.2 months	MI and SON	Active paradigms (MI) and passive paradigms (SON)	Active paradigms:5 blocks; passive paradigms:5 blocks	CW,760 and 830 nm,10 Hz	H17 the prefrontal cortex	5 channels	Time-domain analysis
Si et al. (83)	19 DoC:4 F and 15 M,14 ~ 66 years	10 UWS and 9 MCS	6 TBI, 11 Stroke, 2 Anoxia	63 ~ 4,380 days	MI	Active paradigms	6 questions, 4 blocks per question, 24 blocks in total.	CW,760 and 850 nm,10.2 Hz	PFC, PMC and primary sensorimotor cortex	48 channels	Time-domain analysis
Kempny et al. (36)	16 pDoC:6 F and 10 M,18 ~ 68 years, 10 HC:6 F and 4 M, mean age 40 years	5 VS/UWS and 2 MCS	4 TBI, 5 Anoxic, 7 Stroke	1.8 ~ 80.9 months	MM and MI		MM: 20 blocks MI: 20 blocks	CW,760 and 850 nm,10.24 Hz	The premotor area and supplementary motor area and over the PMC	16 channels	Time-domain analysis
Li et al. (28)	5 DoC:1 F and 4 M, aged 30–49, 6 HC:2 F and 4 M, aged 22–33	5 MCS	3 TBI, 1 Stroke, 1 Anoxia	2 ~ 48 months	MI		5 questions, 4 blocks per question, 20 blocks in total.	CW,690 and 830 nm,28 Hz	PFC, PMC	50 channels	Time-domain analysis
Abdalmalak et al. (126)	1 DoC, M, 75 years old	1 DoC	1 Guillain–Barré syndrome	/	MI		3 questions, 5 blocks per question, 15 blocks in total.	time-resolved functional near-infrared spectroscopy,760 and 830 nm,80 MHZ	The supplementary motor area and the premotor cortex	4 channels	Time-domain analysis
Kurz et al. (127)	2 MCS:1 F,78 years old, 1 M, 31 years old, 10 HC:2 F and 8 M, mean age 26.2 years	2 MCS	1 Wernicke-Encephalopathy, 1 Polytrauma	/	Mental arithmetic tasks		20 blocks	CW,760 and 850 nm,3 Hz	Frontal brain regions	47 channels	Time-domain analysis

TBI, traumatic brain injury; HIE, Hypoxic–ischemic encephalopathy; Vascular, Cerebrovascular events; aSAH, aneurysmatic subarachnoid hemorrhage; aSDH, aneurysmatic subdural hemorrhage; ICH, intracerebral hemorrhage; CH, cerebral hemorrhage; CI, cerebral infarction; CW, continuous wave; DBS, Deep brain stimulation; tDCS, transcranial direct current stimulation; PreM, Premotor; SMA, supplementary motor area; DAN, dorsal attentional network; DMN, default mode network; FPN, frontoparietal network; SEN, sensorimotor network; VAN, ventral attentional network; VIS, visual network; FEF, frontal eye fields; FPA, frontopolar area; DLPFC, dorsolateral prefrontal cortex; PFC, the prefrontal cortex; MM, motor movement; MI, motor imagery; DAN, dorsal attentional network; DMN, default mode network; S1, primary somatosensory cortex and M1, primary motor cortex. PMC, primary motor cortex.

**Table 2:** Summary of ongoing clinical trials of fNIRS registered by WHO at home and abroad in the last 10 years.

Registration number:	Public title	Inclusion criteria	Study type	Observations/treatment measures	Expected end time
ChiCTR2400085830	Quantitative regulation of spinal stimulation characteristics of disorders of consciousness based on photoelectric synchronous brain function detection	Control group A. Meet the diagnostic criteria of chronic disorders of consciousness. B. Age 18 to 60 years old, gender is not limited. C. Consciousness disorder for 3 to 6 months. D. Cerebral cortical structures are relatively intact on at least one side of the brain, and there is no serious damage to brainstem structures. E. Normal or diminished cerebral metabolism, but the metabolism of the brainstem is essentially normal and NIR assessment is appropriate. F. No contraindications to NIR testing, no contraindications to EEG testing, and no cerebral structural abnormalities as determined by the imaging physician. G. Able to cooperate in observing adverse events and efficacy. H. Female patients with a negative pregnancy test. I. Cardiac, pulmonary, hepatic, and renal functions are essentially normal. Expected survival time: >3 months. J. Written informed consent signed by the patient’s legal representative. Treatment group A. Meet the diagnostic criteria of chronic disorders of consciousness and have undergone SCS implantation. B. Age 18 to 60 years old, gender is not limited. C. 3 to 6 months of impaired consciousness and failure of conventional treatment methods to promote awakening. D. Cortical structures are relatively intact on at least one side of the brain, and there is no serious damage to brainstem structures. E. Normal or diminished cerebral metabolism, but the metabolism of the brainstem is essentially normal and NIR assessment is appropriate. F. No contraindications to NIR testing, no contraindications to EEG testing, and no cerebral structural abnormalities as determined by the imaging physician. G. Able to cooperate in observing adverse events and efficacy. H. Female patients with a negative pregnancy test. I. Cardiac, pulmonary, hepatic and renal functions are essentially normal. Expected survival time: >3 months. J. Written informed consent signed by the patient’s legal representative.	Interventional study	Interventional study10 patients underwent Group A stimulation parameter modulation after completion of SCS surgery: frequency 5 Hz, voltage 2 V, pulse width 210 μV, alternating ON (30 s) and OFF (3 min) of SCS, repeated 10 times, and three data collections were completed within one week for each patient.	December 31, 2025
ChiCTR2400083564	Based on functional near infrared spectroscopy, the effect of acupuncture on brain network activation in patients with consciousness disorder after brain injury was studied	(1) The new brain trauma was diagnosed by head imaging and met the evaluation criteria of consciousness disorder. (2) After 3 days of surgical treatment, there are still conscious disorders, and the score of Glasgow Coma Scale (GCS) is less than 13. It is estimated that the ICU stay will be no less than 14 days. At present, the condition is stable, with no serious infection and stable vital signs. (3) Consciousness was clear before injury, and there was no primary disturbance of consciousness and serious cerebrovascular disease. (4) The age is 18 ~ 60 years old. (5) No skull defect. (6) The family members signed the informed consent form and volunteered to participate in the study. (7) No acupuncture treatment was performed in the last 2 weeks. (8) No contraindications to acupuncture: needle sickness, hemophilia, severe skin infection, etc.	Interventional study	Acupuncture	December 31, 2025
ChiCTR2300074202	Modulation of cerebral cortex activity by auditory sensory stimulation in patients with disorder of consciousness: An fNIRS study	Patient 1. Patients meeting the European Society of Neurology guideline PDoC criteria (more than 4 weeks after brain injury). 2. UVS/VS or MCS were diagnosed after 5 CRS-R assessments within 1 week. 3. The cause was clear and the vital signs were stable. 4. MMN amplitude of auditory evoked potential test was >0.6 μV and < 3 μV; 5. Age 18–75 years old. 6. Right-handed. 7. Have not participated in other clinical studies in the past 3 months. 8. Sign an informed consent form signed by a family member or authorized person. Healthy person 1. Match the age of the patient, between 18 and 75 years old, regardless of gender; 2. Good health, no circulatory system, nervous system, mental and metabolic disorders and other diseases; 3. Having full capacity for civil conduct; 4. The subject voluntarily signs the informed consent.	Interventional study	Group: Patient control group placebo Group: Patient intervention group FAST scheme	July 31, 2026
ChiCTR2300072736	Application and construction of outcome prognostic model in prolonged disorders of consciousness patients based on functional near infrared spectroscopy	1. Meet the B6:C7diagnostic criteria for UWS as proposed by Giacino in 2010 for the international diagnosis of MCS in 2002; or have had MCS or UWS in the past and now have EMCS; and normal individuals; 2. Patients without a recent tendency to improve consciousness who have been ill for more than 1 month; 3. No sedative or antiepileptic medication within the last 1 month; 4. No significant cerebral edema and no severe brain atrophy; 5. Age 18–70 years; 6. Normal control or informed consent signed by the patient’s family.	Observational study	Multidimensional stimulus (sensory, motor, cognitive) assessment	December 31, 2025
ChiCTR2300071307	Euroregulatory mechanisms of thalamo-cortical circuit rTMS in consciousness disorders based on multimodal techniques	1. Refer to Wang Zhongcheng Neurosurgery to formulate diagnostic criteria for the formation mechanism of craniocerebral injury coma and the timing of recovery-promoting treatment: (1) There is an obvious history of craniocerebral injury and cerebral hemorrhage, and some patients have a history of brain herniation; (2) Patients with severe craniocerebral injury diagnosed by head CT or MRI and clinical examination (such as observation of pupils, etc.) and admitted to hospital within 24 h after injury; (3) GCS score for coma, coma of more than 6 h after injury or deterioration of consciousness within 24 h after injury and coma again for more than 6 h; (4) Under the age of 70; (5) Survival period >72 h with stable vital signs, case observation for 72 h after surgery, no active intracranial hemorrhage; (6) All are inpatients in the intensive care unit of our hospital, and have medical records; (7) The patient’s family members gave informed consent to the treatment and signed the informed consent form. Those who meet the above diagnostic criteria can be included in the treatment observation cases.	Interventional study	Routine rehabilitation + Transcranial magnetic stimulation (1HZ)	April 30, 2025

## 2 Basic principles of fNIRS

### 2.1 Technical principle fNIRS

**Figure 3:**
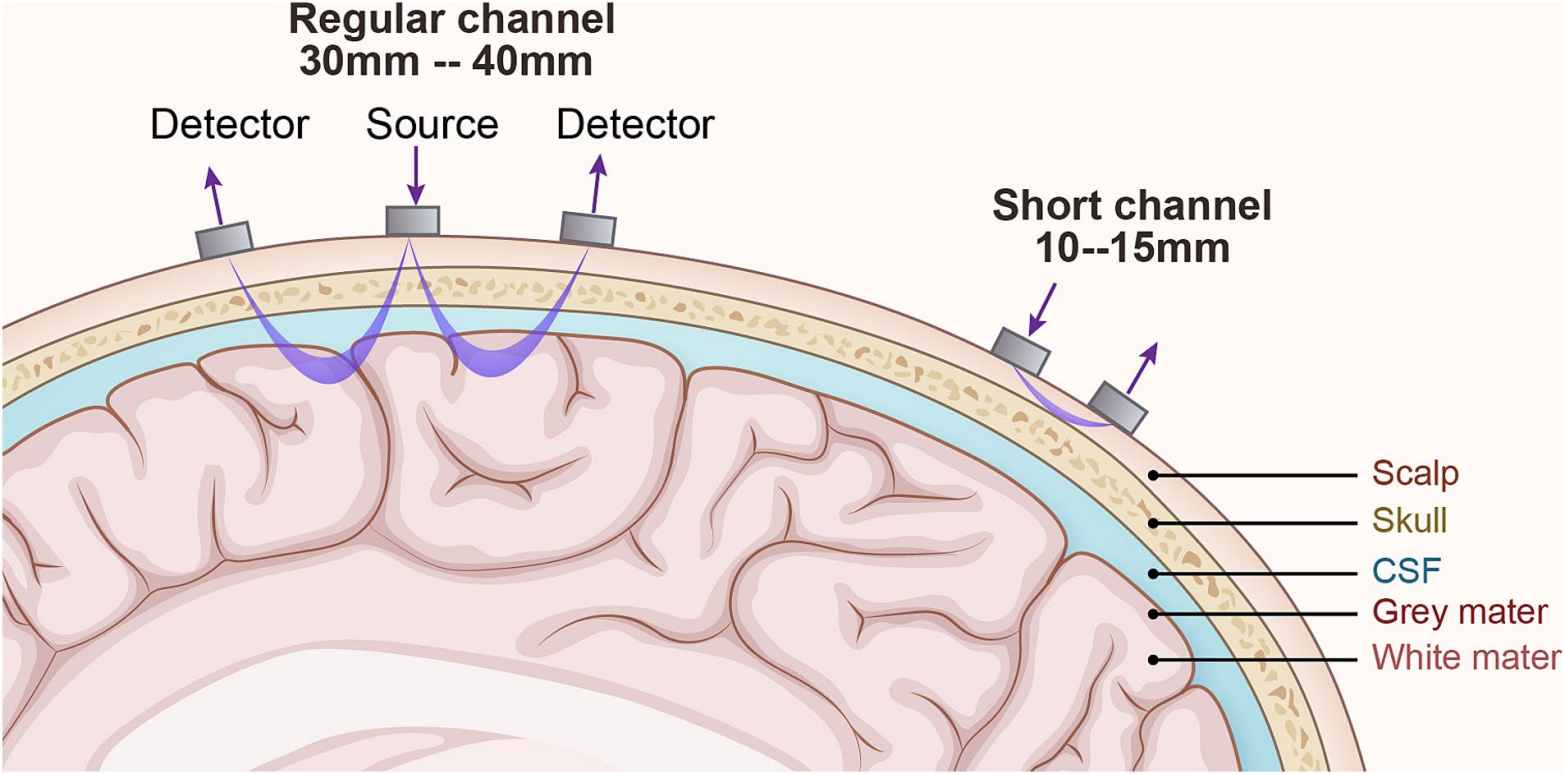
The technical principles of fNIRS. The detector is the receiving light source, and the source is the emitting light source. Generally, the detector and source are 3-4cm for adults and 1–1.5 cm for children. The changes in light intensity of the emitting and receiving light sources respond to changes in blood oxygen concentration in the cerebral cortex and then speculate on neuronal activity.

The light within the 700–900 nm range, referred to as the ‘optical window’, possesses several distinct characteristics: firstly, it can penetrate tissues such as the scalp and skull to reach the intracranial space; secondly, the overall absorption coefficients of chromophores (water, hemoglobin, etc.) are relatively low, ensuring that the light is not entirely absorbed before it exits the scalp and reaches the detector; and lastly, there is a significant difference in the absorption coefficients of HbO and HbR within this range. Consequently, it is possible to select two or more wavelengths and, by leveraging the differences in absorption coefficients between HbO and HbR at these wavelengths, separately determine the concentration changes of HbO and HbR. After near-infrared light is emitted from the source, it passes through the scalp, skull, and cerebrospinal fluid, entering the cerebral cortex where it undergoes absorption, scattering, and refraction before reaching the detector, exhibiting a typical “banana” shape, as shown in Figure 3. The typical fNIRS system consisted of several sources and detectors with a 3 cm source-detector separation (34). During the data analysis process, the modified Beer–Lambert law (MBLL) (35) is applied to infer backward from changes in light intensity to calculate changes in hemodynamic responses, which allows for the deduction of neural activity in the brain, and subsequently, the analysis of the synergistic actions between various brain regions during task execution.

In the field of DoC, fNIRS plays a pivotal role in assessing residual consciousness by measuring changes in hemodynamics and evaluating brain functional connectivity (31). It can also assess brain activity in DoC patients using MI task paradigms (36). Additionally, fNIRS is effective in evaluating treatment efficacy by monitoring oxygenation changes during various therapeutic interventions (32).

### 2.2 fNIRS system

fNIRS is a non-invasive imaging technology that measures blood oxygenation changes in brain tissue, offering significant potential in assessing patients with DoC (37). Key aspects of fNIRS use in DoC include system types, sensor configuration, and data analysis methods, each of which has a profound impact on measurement accuracy, data complexity, and clinical applicability. Table 1 presents a comprehensive overview of the application of fNIRS in patients with DoC. The table is organized into two main sections: Patient Information and Equipment Details. In the Patient Information section, key demographic and clinical details are provided, including age, gender, etiology, and time since onset. Additionally, the paradigms employed in the studies are summarized, encompassing resting-state assessments, active paradigms (e.g., MI tasks), and passive paradigms (e.g., subject’s name). The Equipment Details section outlines the technical aspects of the studies, such as the specific fNIRS systems used, the number of channels employed, and the analytical methods applied. It also includes information on the targeted brain regions selected based on the experimental design and clinical objectives, highlighting the tailored approach for evaluating DoC patients.

### 2.3 fNIRS instrumentation

fNIRS systems can be classified into three main categories: continuous wave (CW), frequency domain (FD), and time domain (TD) systems. CW systems are the most used fNIRS systems. They utilize a constant-intensity near-infrared light source to measure absorption differences across various wavelengths, thus estimating relative concentration changes in HbO and HbR (19). CW systems are relatively simple, low-cost, and allow for rapid data acquisition, making them ideal for bedside monitoring and high-throughput research. However, CW systems measure only relative concentration changes, lacking the capacity to provide absolute concentrations of HbO and HbR. Without information on photon travel time or phase shift, CW systems have inherent limitations in accuracy (38). FD technology employs intensity-modulated near-infrared light, where the incident light intensity is modulated with a sinusoidal wave at a frequency of 100–200 MHz on top of a steady-state value (39). As the incident light propagates through tissue, it experiences attenuation and scattering, leading to a reduction in the amplitude of the high-frequency sinusoidal wave and a phase delay. By detecting the emitted light and demodulating it, amplitude attenuation and phase shift can be utilized to obtain the tissue’s absorption and scattering coefficients. From these optical parameters, the absolute concentrations of oxygenated and deoxygenated hemoglobin in the tissue can be determined (40). FD systems use modulated light sources to measure phase shifts and amplitude variations, enabling the estimation of absolute HbO and HbR concentrations (40). FD systems provide high accuracy and absolute concentration measurements, which are critical for precise hemodynamic assessments in DoC patients. Compared to CW systems, FD systems are more complex and expensive, limiting their widespread application in clinical settings (25). TD systems emit ultrashort light pulses and measure the distribution of photon travel times within tissue, capturing detailed absorption and scattering properties (41). TD systems offer the most precise measurements of absolute concentrations and tissue scattering, making them suitable for in-depth research. TD systems are the most complex and costly, with high data processing demands, making them less feasible for routine clinical use. TD systems and FD systems can provide more detailed information, but their technology is more complex, and the equipment is significantly more expensive than that of CW systems, with lower signal-to-noise ratios, thus limiting their applications (42). The vast majority of near-infrared imaging instruments utilize CW systems. In cases where absolute concentrations of HbO and HbR are required, FD systems offer a higher signal-to-noise ratio and faster acquisition speed compared to TD systems, but it is slightly inferior to TD systems in distinguishing between scattering and absorption effects and in terms of depth resolution (16, 42). A comparative analysis of three technologies is presented in Table 3.

**Table 3:** Comparison of fNIRS systems with three different modulation techniques.

	Continuous wave (CW)	Frequency domain (FD)	Time-resolved (TD)
Measurement signal	Relative optical intensity change	Relative optical intensity and phase change	Time-of-flight curve and relative optical intensity change
Functional parameter	Relative Hb	Absolute Hb	Absolute Hb
SNR	High	Moderate	Low
Depth resolution	Low	High	Moderate
Instrument size	Some bulky, some small	Bulky	Bulky
Instrument cost	Some low, some high	Very high	Very high
Level of application maturity	High	Moderate	Low

Hb, Hemoglobin; SNR, Signal-to-Noise Ratio.

### 2.4 Arrangement of fNIRS optodes

The number and placement of fNIRS sensors (light sources and detectors) significantly impact measurement accuracy and spatial resolution. A higher number of fNIRS optodes enables greater cortical coverage and precision, but it also increases data volume and complexity. Configurations typically range from a few to dozens of optodes, depending on the areas of interest (20).

The International 10–20 system is widely adopted in fNIRS studies for its standardized and consistent approach to optode placement, ensuring optimal coverage of critical cortical regions such as the prefrontal, parietal, and temporal lobes. Approximately 70% of fNIRS studies in DoC research utilize montages based on the 10–20 system due to its reliability and compatibility with existing neuroimaging methods (41, 43). This system facilitates cross-study comparisons and provides a foundation for interpreting functional brain activity in DoC patients. However, alternative optode configurations have also been employed to meet specific research and clinical needs. High-density optode grids, for instance, offer improved spatial resolution, allowing for more detailed mapping of cortical activation patterns. Despite their advantages, these configurations often require more complex data processing and are less portable, making them challenging to implement in bedside settings (16). Conversely, sparse optode arrangements prioritize portability and ease of application, particularly in clinical environments. While these setups are advantageous for rapid assessments, they may compromise spatial resolution and limit the ability to detect activity in deeper cortical regions (44). Hybrid approaches, which combine high-density grids for key cortical areas with sparse arrangements for peripheral regions, aim to balance these trade-offs (45). These variations in optode placement underscore the importance of selecting appropriate configurations based on study objectives and patient conditions. The flexibility of fNIRS in accommodating different sensor montages highlights its adaptability and potential for diverse clinical and research applications in the DoC field.

### 2.5 Data analysis methods in fNIRS

Theoretically, fNIRS data would solely encompass the blood oxygenation signal components induced by experimental tasks. However, in practical data acquisition processes, various types of noise are inevitably superimposed on the data. The sources of noise in fNIRS data are diverse: the measurement system, physiological origin, and head/body motion (46). Below are several common types of fNIRS noise and their removal methods. Firstly, systemic physiological changes affect cerebral hemodynamics. The primary physiological factors that can confound fNIRS measurements include fluctuations in the partial pressure of carbon dioxide (PaCO2) (around 0.2–0.6 Hz), systemic blood pressure (around 0.1 Hz), and alterations in heart rate (around 0.8–1.6 Hz) (47, 48). Secondly, signal drift is a pervasive type of noise in fNIRS brain imaging signals, typically manifesting as slow fluctuations over extended periods. Signal drift can alter the baseline of fNIRS signals. The sources of fNIRS brain imaging signal drift are complex and include interference noise during the signal acquisition process of the imaging system (such as the effects of the machine gradually warming up during operation), as well as the slow head movements of the subject that are imperceptible to the naked eye during the experiment. Thirdly, head motion and motion artifacts (MA) are a common type of noise in fNIRS signals; subject head movements or limb activities may lead to poor contact between the optodes and the scalp, often reflected as large jumps in the signal. Generally, fNIRS has a low signal-to-noise ratio, and without “denoising,” it will directly affect the subsequent calculation of individual hemodynamic response indicators and may even lead to false group activations. Bandpass filtering can be used to remove high-frequency cardiac signals and low-frequency drifts in the optical signal (49). It is crucial to pay special attention to not disrupt the frequencies related to the experimental tasks during the filtering process. Otherwise, the expected activation results cannot be obtained. Nevertheless, the frequency range of respiratory and Mayer wave oscillations coincides with that of the hemodynamic response, thus precluding their simple elimination via bandpass filtering (50). Due to the abundance of capillaries in superficial tissues such as the scalp, skull, and meninges, physiological fluctuations such as respiration and heartbeat, as well as task-related autonomic nervous activities, can cause changes in the concentration of hemoglobin in these capillaries. When near-infrared light passes through these superficial tissues, changes in the concentration of hemoglobin in the superficial vessels also lead to variations in the optical attenuation of fNIRS. Superficial physiological noise has a significant impact on fNIRS signals (51). Currently, the short-channel subtraction has been considered the ‘gold standard’ to reduce the responses from the extracerebral tissue in fNIRS data (52). The optical path of short interoptode channels is relatively shallow, generally passing only through superficial tissues without reaching the cerebral cortex. Therefore, it is generally believed in the academic community that short interoptode channels primarily record superficial physiological noise. This method employs additional short-distance fNIRS channels to record the superficial physiological noise and subsequently removes it from the signal (53). Alternative approaches are data-driven signal processing methods that decompose the fNIRS signals into its brain and systemic components, e.g., principal component analysis (PCA) (46). In previous publications, PCA has been widely used to remove head motion and motion artifacts in fNIRS data (54–56). In DoC patients, sensor placement strategies that reduce susceptibility to movement artifacts are particularly valuable given the limited ability of these patients to remain still (57, 58).

Time-domain analysis evaluates changes in light intensity over time to correlate hemodynamic responses with specific tasks or stimuli. In DoC research, this method is widely used to assess brain responses to external stimuli, helping to detect residual cognitive activity. For instance, Pipeline 1 involves preprocessing steps such as motion artifact removal and signal detrending, followed by task-based averaging to identify significant activation. Pipeline 2 incorporates additional steps like deconvolution modeling to enhance temporal resolution, allowing for the differentiation of overlapping hemodynamic responses. A recent meta-analysis revealed that 70% of studies employed Pipeline 1 due to its simplicity, while 30% utilized Pipeline 2 for more complex paradigms. Pipeline 1 is advantageous for rapid analysis but is limited in detecting subtle temporal changes, whereas Pipeline 2 provides greater accuracy at the cost of computational complexity (16, 44). Frequency Domain Analysis examines the power spectral density of fNIRS signals, capturing brain wave activities such as alpha and theta waves, as well as low-frequency oscillations (0.01–0.1 Hz) (45). Frequency domain analysis is useful for understanding neural networks and functional connectivity changes in DoC patients. By combining multi-channel data, spatial analysis generates maps of brain activity, allowing for the visualization of functional distribution across different cortical areas (59). Spatial analysis generates brain activity maps by combining multi-channel data to visualize functional distribution across cortical areas. Techniques such as t-maps, derived from statistical comparisons of task versus baseline HbO levels, are commonly employed to identify active regions. Additionally, functional nodes and network hubs are identified through clustering algorithms, offering insights into the spatial organization of neural networks. This approach has been used to map activity in regions like the PFC, SMA, and FPA, providing a detailed understanding of localized brain functions (45, 60). This approach provides valuable insights into the regional brain activity patterns in DoC patients. Functional connectivity analysis assesses the synchronized activity across brain regions, revealing the organization of functional networks (16). Changes in functional connectivity offer essential information on the integrity of neural networks, making this approach particularly useful in DoC assessments.

In summary, fNIRS is a promising tool for DoC assessment, with unique advantages such as non-invasiveness, portability, and real-time monitoring capabilities. This discussion highlights several gaps in current research on fNIRS for DoC applications and potential directions for future studies: Current DoC studies predominantly utilize CW systems, with limited exploration of FD and TD systems in clinical settings. Future studies should investigate FD and TD systems to improve accuracy and enable absolute concentration measurements. Furthermore, increasing sensor density and optimizing placement strategies for critical cortical regions, such as the PFC and SMA, could enhance spatial resolution and reduce MA. Finally, adopting multidimensional analyses, including spatial and connectivity measures, may uncover complex network dynamics in DoC patients, advancing diagnostic and therapeutic strategies.

## 3 Clinical applications of fNIRS in DoC

Figure 2 provides a comprehensive summary of the application of fNIRS in the field of DoC, highlighting its current and future roles in clinical and therapeutic contexts. The figure is divided into four sections, including a schematic representation of fNIRS applications in DoC patients. In the upper-left green frame, the focus is on the diagnostic and assessment capabilities of fNIRS for DoC patients. This includes detecting residual consciousness during resting states and evaluating brain activation during task states. These applications underscore the potential of fNIRS as a non-invasive tool for identifying preserved cognitive and motor functions in patients with DoC. The lower-left blue frame addresses the main limitations and prospects of fNIRS. Key challenges include limited spatial resolution, signal interference from extracerebral tissues, and the need for advanced signal processing techniques. Future advancements, such as improved sensor designs and multimodal integration with other imaging modalities, are anticipated to enhance the accuracy and clinical utility of fNIRS. In the yellow frame on the right, the therapeutic applications and future potential of fNIRS are explored. Emphasis is placed on its integration with neuromodulation techniques, such as DBS and SCS, to monitor and optimize therapeutic outcomes. Additionally, the development of BCIs presents a promising direction for combining fNIRS with advanced neurotechnology to refine treatment strategies and improve patient prognoses.

**Figure 4:**
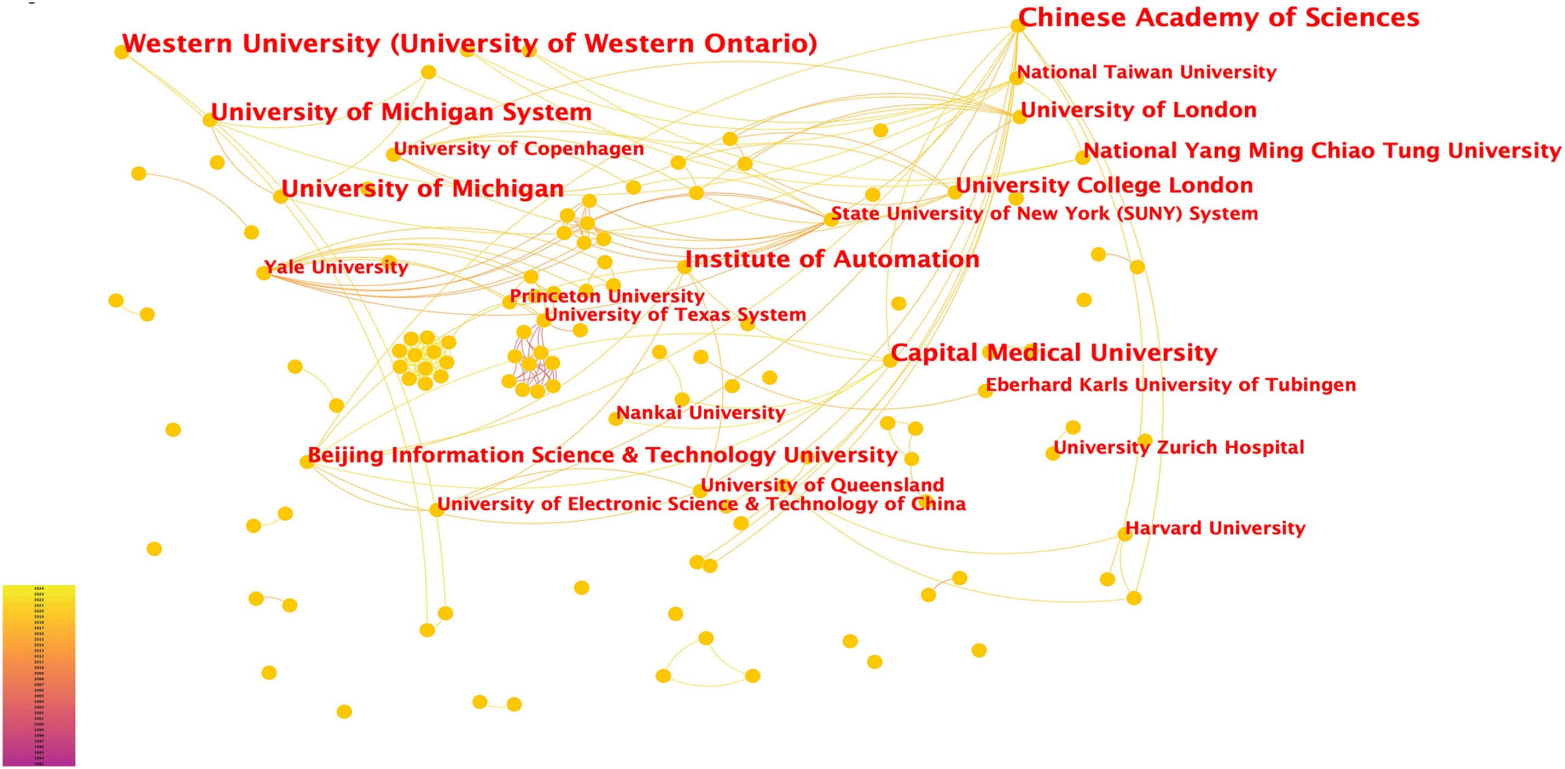
Institutional Contributions to fNIRS Research. Each node in the figure represents a research institution. The font size of the institution name corresponds to the number of articles published, with larger font sizes indicating higher publication output. The density of connections between nodes reflects the level of collaboration between institutions, with thicker or denser connections signifying stronger cooperative relationships.

**Figure 5:**
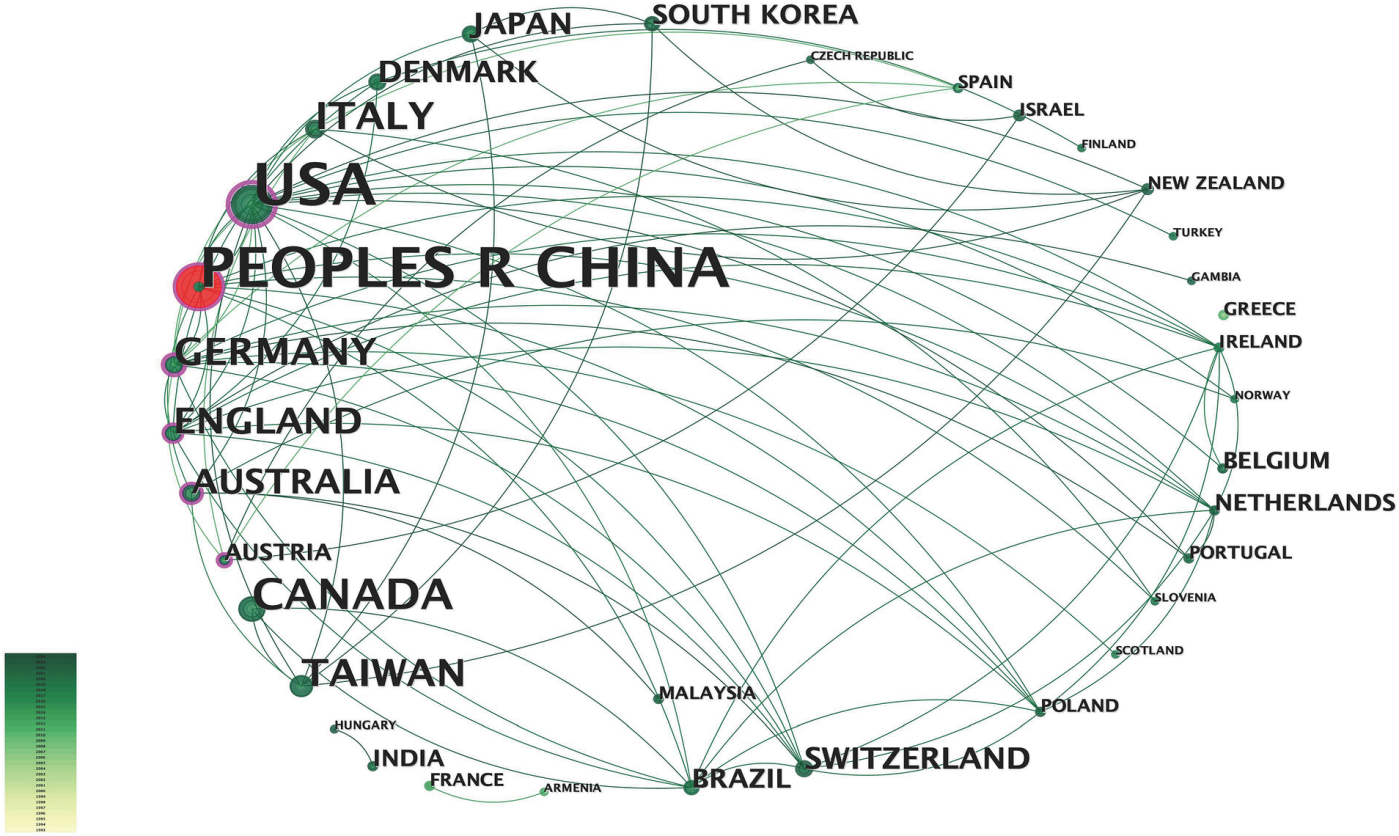
Countries or regions contributing to fNIRS research. The larger the text in the node, the greater the number of articles sent by the country. The node center red indicates a dramatic increase in the number of posts from that country or region.

From 1993 to 2024, 243 institutions have participated in fNIRS research (Figure 4). Research on fNIRS spans 35 countries, with the United States leading in publication output: the earliest U.S. publications date back to 2002, with 40 articles. The U.S. also holds the strongest intermediary position in collaborative networks. China ranks second, with 35 publications. Although China’s engagement in fNIRS research began later than the U.S., the minimal difference in publication volume underscores China’s growing interest and active involvement in fNIRS research (Figure 5). In China, fNIRS research is mainly conducted at leading cognitive psychology institutions such as Beijing Normal University. fNIRS research involves psychology, sociology, aerospace, and other related cognitive-behavioral research (61, 62). In the medical field, fNIRS is increasingly used in psychiatry, pain management, rehabilitation, and neurosurgery. Its applications range from disease diagnosis to supporting the development of brain-computer interface technologies, demonstrating its growing importance as an auxiliary clinical diagnostic tool (63–65). These statistical diversities of the literature represent the current research hotspots of fNIRS in the field of DoC, highlighting the expanding role of fNIRS in bridging cognitive science and clinical medicine, paving the way for future research and practice advances.

### 3.1 Monitoring and assessment of residual consciousness

Numerous studies suggest that the human brain operates within a sophisticated network for functional connectivity and integration. This network exhibits a delicate balance between spontaneous segregation and integration during neuronal activity, which is essential for cognitive, perceptual, or motor signaling (66, 67). Its strength is typically assessed using correlation coefficients and quantified using graph-theoretic topological analysis of resting-state data (68, 69).

In patients with DoC, the functional connectivity of the resting-state brain network is significantly disrupted, with diminished strength compared to that of healthy individuals (70). Specifically, the functional connectivity in the frontal lobes, particularly the frontal pole region and the right dorsolateral prefrontal cortex, is severely impaired in patients with MCS (71–73). CRS-R remains the clinical gold standard for distinguishing between VS and MCS (74). However, behavioral assessments based on the CRS-R are prone to subjective, influenced by the clinical assessor’s understanding of DoC criteria and their application of quantitative scales. Such variability may result in biased or erroneous diagnoses (6, 75, 76) To address these limitations, objective assessment tools are essential for accurately evaluating the sensory, motor, and cognitive functions of DoC patients and for predicting neurological recovery (76, 77). These tools provide a crucial complement to subjective assessments, enabling more reliable and standardized evaluation methods. Experimental paradigms for detecting and assessing levels of consciousness typically fall into two categories: resting-state brain functional connectivity analyses and task-based brain response analyses, the latter of which includes both passive and active paradigms.

#### 3.1.1 Resting-state paradigm

The fNIRS resting-state paradigm is primarily utilized to evaluate the functional brain state of patients with DoC. It allows clinicians to more accurately assess patients’ level of consciousness, thereby supporting informed clinical management and decision-making. After mitigating noise from physiological artifacts, such as respiration and heartbeat, resting-state fNIRS data analysis can produce robust correlation maps and theoretical indicators. These indicators represent functional connectivity between brain regions, offering valuable insights into spontaneous brain activity in the absence of specific tasks and its relationship with disease (78, 79).

Liu et al. demonstrated the efficacy of resting-state fNIRS in detecting residual neurofunctional connectivity, confirming its effectiveness in identifying the status of DoC patients (80). Similarly, Urquhart et al. highlighted the feasibility of using fNIRS in various functional connectivity metrics, showing how physical activity influences cortical networks across different frequency bands (81). Furthermore, He et al. assessed residual consciousness in 18 DoC patients using resting-state fNIRS and applied graph-theoretic analyses to improve classification accuracy. Their findings provided valuable insights into the diagnosis of DoC patients and enhanced understanding of the pathological mechanisms underlying DoC, thereby advancing prospects for neurological recovery (31).

#### 3.1.2 Active paradigm

Using the fNIRS, patients with DoC can be assessed for residual consciousness through passive tasks and active commands-based paradigms. In clinical practice, fNIRS has demonstrated its value in identifying patients with locked-in syndrome or impaired consciousness, thereby expanding its clinical applicability (12). “One approach involves measuring changes in prefrontal blood oxygenation in response to auditory stimuli. For instance, patients are asked to listen to two simple questions and imagine answering ‘yes’ or ‘no.’”. Results showed that some of the MCS patients and one patient with UWS exhibited blood oxygenation changes consistent with patterns observed in healthy controls, indicating residual verbal comprehension and volitional expression (42). Another study demonstrated that three out of five MCS patients produced with one patient answering all questions accurately using this method. This study confirms the feasibility of the fNIRS in detecting residual cognitive abilities in DoC patients through active command-driven MI (28). fNIRS technology provides a non-invasive and reliable means to measure neuronal activity and assess stimulus–response relationships in DoC patients, offering critical insights into their potential for neurological recovery. This capability can significantly influence their future treatment strategies and clinical management.

Patients with different types of docs are instructed to imagine performing simple or complex movements, such as speaking or shaking a fist, to assess their motor function and recovery potential using MI tasks under active command, which can be divided into visual and motor tasks. These tasks, categorized as visual or motor, are closely linked to the corticomotor system, as demonstrated by visual and kinesthetic imagery (82). One study found that some MCS could follow commands to imagine shaking a fist with either their left or right hand. The cortical hemodynamic changes measured by fNIRS in these patients were consistent with those observed in healthy controls (36). Another study compared the hemodynamic responses of the MCS and UWS patients during imagery tasks, revealing that the MCS group exhibited higher hemodynamic responses and better CRS-R scores, whereas the UWS group showed no significant changes (83). These findings suggest that MCS patients have greater potential for neurological recovery. Furthermore, fNIRS has proven to be a convenient and effective tool for detecting residual communication and awareness in DoC patients.

#### 3.1.3 Passive paradigm

fNIRS can assess different degrees and types of Docs by detecting cognitive or perceptual responses to stimuli that do not require active participation or response. These stimuli may include touch, images, and sounds. For instance, one study used fNIRS to measure prefrontal blood oxygenation changes in patients with prefrontal blood oxygen changes in patients with VS, MIS, and MCS as they listened to recordings of their names and unfamiliar names (84).

### 3.2 Quantitative evaluation of neuromodulation therapy effects

Neuromodulation therapy plays a pivotal role in treating refractory neurological disorders such as epilepsy, Parkinson’s disease, and depression (85, 86). This technology encompasses both non-invasive and implantable approaches, delivering electromagnetic stimulation or chemical agents to targeted regions of the management of DoC, SCS and DBS are the most commonly employed neuromodulation techniques (87).

#### 3.2.1 Deep brain stimulation

The precise mechanism of action of DBS remains unclear, but it is widely believed to regulate neurotransmitter release, inhibit abnormal neural activity, stimulate synapse activity and neural remodeling, and alleviate symptoms of brain dysfunction (88). The first documented use of DBS in treating DoC was reported in 1969, demonstrating its potential to restore conscious arousal and pathways following loss of consciousness (89, 90).

DBS is a critical tool in neuromodulation and monitoring cognitive function during stimulus on and off periods is essential to evaluate its modulatory effects. A recent study utilized fNIRS to measure blood flow changes in the frontal, parietal, and occipital lobes. The findings revealed that global communication efficiency correlated significantly with the CRS-R index (slope = 57.384, *p* < 0.05, r = 0.483), while no significant relationship was observed between changes in communication strength across six brain lobes and the CRS-R index (91). The integration of fNIRS into DBS therapy provides valuable insights into its impact on neurological restoration and recovery in DoC patients. This combination underscores the potential of fNIRS-based functional connectivity analysis as a powerful quantitative assessment tool in neuromodulation research and clinical applications.

#### 3.2.2 Spinal cord stimulation

SCS has been studied using the fNIRS passive paradigm. Unlike fMRI, fNIRS is unaffected by metallic implants, such as deep or spinal cord stimulators, making it suitable for assessing real-time activity changes during neuromodulation therapy in patients with DoC. This capability provides crucial clinical guidance for regulating the frequency and duration of stimulation during SCS therapy. Zhang et al. reported a significant increase in cerebral blood volume in the prefrontal and occipital cortex when SCS was applied at a stimulation interval of 30 s. Their findings suggest that optimizing stimulation intervals can promote neurological function recovery in DoC patients, offering robust evidence and a feasible research paradigm for further quantitative assessment of treatment effects (33). Si et al. measured hemodynamic changes in the prefrontal and occipital lobes of DoC patients at varying frequencies using SCS. They observed that high-frequency SCS at 70 and 100 Hz significantly enhanced the cerebral hemodynamic response in the prefrontal region, with SCS at 70 Hz notably improving functional connectivity between the prefrontal and occipital lobes (92). In conclusion, fNIRS-based visualization enhanced the standardization of SCS therapy and supports neurological recovery in DoC patients.

Additionally, fNIRS-based visualization enhances the standardization of SCS therapy and supports neurological recovery in DoC patients. Additionally, closed-loop neuromodulation techniques such as repetitive transcranial magnetic stimulation (rTMS) and vagus nerve stimulation (VNS) are widely employed in DoC treatment. However, research combining these techniques with fNIRS remains limited. Future studies exploring the use of fNIRS to evaluate the therapeutic effects of rTMS and VNS in DoC may provide valuable insights into the advancement of neuromodulation strategies.

### 3.3 BCI

BCI technology enables direct interaction between the brain and external devices by deciphering neural activity, serving as a means of communication and neurological function recovery. It has been applied in various fields, including communication, artificial intelligence, and biomedicine. In recent years, fNIRS-based BCI has shown significant potential in facilitating communication for patients with DoC. Natio et al. demonstrated the feasibility of using fNIRS to analyze cerebral blood flow, enabling communication with amyotrophic lateral sclerosis patients in complete locked-in syndrome. This study highlights the potential of fNIRS in establishing effective BCI systems (93). Notably, fast and efficient fNIRS-BCI communication has been achieved in as little as 2 s (94).

MI and mental arithmetic are the two most common paradigms in fNIRS-based BCI applications. The MI paradigm is particularly suitable for patients with severe physical impairments, as it does not require intact thalamocortical tracts (95). Linguistic imagery tasks, where patients can imagine speaking sentences, words, or numbers, are also employed to evaluate language-related brain activity and assess nerve conduction, linguistic function, and prognosis (84, 96, 97). fNIRS has been used to measure blood oxygenation changes in the frontotemporal lobes of doC patients imagining themselves uttering a sequence of numbers. Some MCS patients exhibited blood oxygenation patterns consistent with those of healthy controls, suggesting residual memory and language production abilities, as well as potential for rehabilitation (98). Integrating fNIRS with EEG enhances BCI performance, creating a weighted assessment system capable of stable long-term monitoring (99). Mental arithmetic, involving simple calculations without external aids, is another widely used paradigm. Since the prefrontal cortex is less prone to data noise due to minimal hair interference, it is often the preferred region for fNIRS studies (100, 101). Mental arithmetic has been successfully applied in BCI research with both brain-injured patients and healthy controls (100).

The fNIRS-based BCI classification approach currently lacks the consistency needed to meet the performance standards required for practical, real-world applications. A “real BCI” refers to a system designed for dynamic, real-world environments that demands high classification accuracy (typically >80%), robustness across users and settings, sufficient information transfer rates, and ease of use. These criteria are critical for reliable applications in clinical and assistive technologies. Recent research has demonstrated promising advancements in this area. Zhang et al. developed a convolution-based conditional generative adversarial network (CGAN) with an improved Inception-ResNet architecture for fNIRS-BCI classification. Their method, tested on MA and mental singing (MS) tasks, achieved a maximum accuracy of 92%, showcasing its potential for high-precision applications (102) Similarly, Jinuk et al. utilized a deep convolutional neural network to distinguish between idle-state and mental arithmetic tasks, achieving classification accuracies exceeding 85%. This approach reduced the need for extensive calibration sessions, thereby improving the practicality of subject-independent fNIRS-BCI systems (103). In addition to widely used machine learning algorithms such as support vector machines (SVM) and linear discriminant analysis (LDA), channel selection methods based on positive Z-scores have been employed to enhance fNIRS-BCI performance. The Z-score method significantly improved classification accuracies, achieving 87.2 ± 7.0%, 88.4 ± 6.2%, and 88.1 ± 6.9% (*p* < 0.0167) for left MI vs. rest, right MI vs. rest, and mental arithmetic vs. rest, respectively. This approach was further validated using an open-access dataset of 17 subjects (104).

BCI systems have been extensively applied to detect cognitive functioning and consciousness in patients with DoC. For example, Guger et al. reported that BCI systems enable efficient and effective communication in patients with locked-in syndrome (105). Additionally, EEG-BCI systems have been used to detect mood alterations and command-following abilities in one-third of DoC patients (106). While the utility of BCI systems in neurological applications is well-documented, research specifically on fNIRS-based BCI systems for DoC patients remains limited. Preliminary findings from other populations suggest that fNIRS-BCI technology could provide valuable insights into brain function. However, its feasibility and effectiveness in supporting prognostic judgment and clinical decision-making for DoC patients require further validation. Nevertheless, the non-invasive nature, portability, and cortical activity assessment capabilities of fNIRS-BCI systems position them as promising tools for aiding functional and cognitive recovery in DoC patients. Continued research in this field could establish a robust foundation for integrating fNIRS-BCI into clinical practice.

## 4 Limitations and prospects

### 4.1 Limitations

fNIRS is an innovative neuroimaging technique capable of assessing residual consciousness and potential neural functions in patients with DoC. By utilizing various near-infrared brain imaging paradigms or tasks, fNIRS can explore cortical structures and neural network restoration, providing valuable guidance for diagnosis, evaluation, and treatment (107, 108). However, several limitations and challenges remain. These include issues with signal quality, spatial resolution, and the lack of standardized protocols, all of which require further investigation and refinement for clinical use. Technical challenges in fNIRS data collection often arise from craniocerebral injuries, such as the effects of intracranial hemorrhages on imaging and poor signal quality due to suboptimal contact between cranial surgical probes and the scalp. Additionally, structural changes in the brain may further degrade signal quality in functional regions (109, 110). Furthermore, fluctuations in alertness, awareness, and concentration in DoC patients present another challenge, complicating the reliability of fNIRS measurements.

### 4.2 Prospects

Among neuroimaging modalities for DoC detection, EEG and fMRI are more commonly used. EEG offers high temporal resolution and has advanced task-based paradigms for assessing and classifying DoC patients, while fMRI provides high spatial resolution and unique insights into functional brain networks. However, fNIRS has emerged as a promising tool for DoC assessment, offering moderate spatial and temporal resolution, superior portability, and cost-effectiveness. Future research in the following areas could enhance its utility.

#### 4.2.1 Improvement of technical precision

Most fNIRS research has focused on detecting brain activity through sensorimotor activity in MI paradigms. However, its sensitivity for detecting task-related activation in single-subject data remains inadequate for clinical applications. Many studies rely on simpler, less sensitive continuous-wave devices. Advancing the use of high-channel-density frequency-domain and time-domain fNIRS systems could enable the capture of clinically meaningful brain responses at higher resolutions. Developing sensitive fNIRS markers for MI, particularly in the sensorimotor cortex, which is easily accessible via scalp-based sensors, would be invaluable for DoC patients.

#### 4.2.2 Multi-modal fusion

Multimodal brain imaging can address the limitations of single modalities by extracting complementary features. Integrating fNIRS with other neuroimaging methods, such as EEG, could provide a more comprehensive and accurate assessment of DoC. Advanced machine learning techniques, including artificial neural networks, could further enhance the sensitivity of fNIRS (111). Recent studies combining fNIRS and EEG for BCI applications have shown that these techniques can meet clinical standards for DoC assessment. The consistent and complementary nature of EEG and fNIRS signals—EEG offering high temporal resolution and fNIRS providing insights into neurovascular coupling—has proven effective. For instance, coupling between fNIRS oxygenated hemoglobin (0.07–0.13 Hz) and EEG band power (1–12 Hz) in the frontal region has been shown to predict consciousness recovery following acute brain injury. Such findings highlight the potential of fNIRS-EEG as a valuable addition to multimodal neuromonitoring (112, 113). Some studies use fMRI to improve the modeling of fNIRS-collected brain data to provide an initial roadmap and for fMRI researchers to add fNIRS experimental assays to their study subjects (114). Mixed-modality imaging may become the future standard for assessing disorders of consciousness.

#### 4.2.3 Large-scale research

Understanding the relationship between fNIRS signals and patients’ consciousness states requires larger datasets. Different types of DoC may exhibit distinct fNIRS signal patterns, necessitating extensive clinical studies to classify and validate these differences. Large-scale research will be crucial for verifying the validity and feasibility of fNIRS as a reliable tool for detecting and evaluating consciousness in DoC patients.

#### 4.2.4 fNIRS-based hyperscanning

Hyperscanning, which allows simultaneous recording of neural signals from two or more participants, was first applied to fNIRS in 2012 to study neural activation during social interactions (115). For example, eye contact between individuals activated relevant neural systems that could not be captured under static conditions. The central subcentral gyrus (BA43) was selectively activated during eye contact, demonstrating connections to facial, linguistic, sensorimotor, and executive functions. This study provided insights into the continuous, bidirectional flow of social interactions. Future fNIRS research could focus on sensory processes underlying coherent neural activity during real-time, spontaneous human interactions, offering new avenues for understanding social and neural dynamics (116).

## 5 Conclusion

This review highlights recent advances in the application of fNIRS for DoC while addressing its current challenges and limitations. As an emerging neuroimaging technique, fNIRS provides a unique capability to explore residual consciousness and potential neural functions in DoC patients, offering valuable guidance for diagnosis, prognostic assessment, and therapeutic management. However, significant challenges remain, including issues with signal quality, spatial resolution, standardized data analysis, and its limited ability to elucidate specific mechanisms of neural recovery. Addressing these challenges will require continued research and technological development.

The integration of multiple imaging modalities to jointly assess consciousness states presents a promising avenue for improving clinical diagnosis and prognosis. fNIRS stands out as a non-invasive, portable, and reliable method for assessing neural activity and evaluating therapeutic outcomes in DoC patients. Its ability to capture real-time brain function changes positions it as a critical tool for guiding treatment strategies and monitoring the efficacy of neuromodulation therapies. Nevertheless, further research is essential to enhance its accuracy, spatial resolution, and integration with other neuroimaging techniques, thereby unlocking its full clinical potential.

## Footnotes

^1^ https://trialsearch.who.int/

## References

[1] van Erp WS Lavrijsen JC van de Laar FA Vos PE Laureys S Koopmans RT. The vegetative state/unresponsive wakefulness syndrome: a systematic review of prevalence studies. Eur J Neurol. (2014) 21:1361–8. doi: 10.1111/ene.12483, PMID: 25039901

[2] Multi-Society Task Force on PVS. Medical aspects of the persistent vegetative state (1). N Engl J Med. (1994) 330:1499–508. doi: 10.1056/NEJM199405263302107 7818633

[3] Bruno MA Vanhaudenhuyse A Thibaut A Moonen G Laureys S. From unresponsive wakefulness to minimally conscious PLUS and functional locked-in syndromes: recent advances in our understanding of disorders of consciousness. J Neurol. (2011) 258:1373–84. doi: 10.1007/s00415-011-6114-x, PMID: 21674197

[4] Laureys S Celesia GG Cohadon F Lavrijsen J León-Carrión J Sannita WG. Unresponsive wakefulness syndrome: a new name for the vegetative state or apallic syndrome. BMC Med. (2010) 8:68. doi: 10.1186/1741-7015-8-68, PMID: 21040571 PMC2987895

[5] Giacino JT Ashwal S Childs N Cranford R Jennett B Katz DI. The minimally conscious state: definition and diagnostic criteria. Neurology. (2002) 58:349–53. doi: 10.1212/WNL.58.3.349, PMID: 11839831

[6] Giacino JT Schnakers C Rodriguez-Moreno D Kalmar K Schiff N Hirsch J. Behavioral assessment in patients with disorders of consciousness: gold standard or fool's gold? Prog Brain Res. (2009) 177:33–48. doi: 10.1016/S0079-6123(09)17704-X, PMID: 19818893

[7] Owen AM. The search for consciousness. Neuron. (2019) 102:526–8. doi: 10.1016/j.neuron.2019.03.024, PMID: 31071287

[8] Kondziella D Bender A Diserens K van Erp W Estraneo A Formisano R. European academy of neurology guideline on the diagnosis of coma and other disorders of consciousness. Eur J Neurol. (2020) 27:741–56. doi: 10.1111/ene.14151, PMID: 32090418

[9] Stender J Gosseries O Bruno M-A Charland-Verville V Vanhaudenhuyse A Demertzi A. Diagnostic precision of PET imaging and functional MRI in disorders of consciousness: a clinical validation study. Lancet. (2014) 384:514–22. doi: 10.1016/S0140-6736(14)60042-8, PMID: 24746174

[10] Harrison AH Connolly JF. Finding a way in: a review and practical evaluation of fMRI and EEG for detection and assessment in disorders of consciousness. Neurosci Biobehav Rev. (2013) 37:1403–19. doi: 10.1016/j.neubiorev.2013.05.004, PMID: 23680699

[11] Wang J Gao X Xiang Z Sun F Yang Y. Evaluation of consciousness rehabilitation via neuroimaging methods. Front Hum Neurosci. (2023) 17:1233499. doi: 10.3389/fnhum.2023.1233499, PMID: 37780959 PMC10537959

[12] Hosni SM Borgheai SB McLinden J Shahriari Y. An fNIRS-based motor imagery BCI for ALS: a subject-specific data-driven approach. IEEE Trans Neural Syst Rehabil Eng. (2020) 28:3063–73. doi: 10.1109/TNSRE.2020.3038717, PMID: 33206606

[13] Edlow BL Claassen J Schiff ND Greer DM. Recovery from disorders of consciousness: mechanisms, prognosis and emerging therapies. Nat Rev Neurol. (2021) 17:135–56. doi: 10.1038/s41582-020-00428-x, PMID: 33318675 PMC7734616

[14] Essenpreis M Cope M Elwell CE Arridge SR van der Zee P Delpy DT. Wavelength dependence of the differential pathlength factor and the log slope in time-resolved tissue spectroscopy. Adv Exp Med Biol. (1993) 333:9–20. doi: 10.1007/978-1-4899-2468-1_2, PMID: 8362674

[15] Fantini S Franceschini MA Fishkin JB Barbieri B Gratton E. Quantitative determination of the absorption spectra of chromophores in strongly scattering media: a light-emitting-diode based technique. Appl Opt. (1994) 33:5204–13. doi: 10.1364/AO.33.005204, PMID: 20935909

[16] Scholkmann F Kleiser S Metz AJ Zimmermann R Mata Pavia J Wolf U. A review on continuous wave functional near-infrared spectroscopy and imaging instrumentation and methodology. NeuroImage. (2014) 85:6–27. doi: 10.1016/j.neuroimage.2013.05.004, PMID: 23684868

[17] Hong K-S Ghafoor U Khan MJ. Brain–machine interfaces using functional near-infrared spectroscopy: a review. Artif Life Robot. (2020) 25:204–18. doi: 10.1007/s10015-020-00592-9

[18] Cheng X Sie EJ Boas DA Marsili F. Choosing an optimal wavelength to detect brain activity in functional near-infrared spectroscopy. Opt Lett. (2021) 46:924–7. doi: 10.1364/OL.418284, PMID: 33577549

[19] Boas DA Elwell CE Ferrari M Taga G. Twenty years of functional near-infrared spectroscopy: introduction for the special issue. NeuroImage. (2014) 85:1–5. doi: 10.1016/j.neuroimage.2013.11.033, PMID: 24321364

[20] Cui X Bray S Reiss AL. Functional near infrared spectroscopy (NIRS) signal improvement based on negative correlation between oxygenated and deoxygenated hemoglobin dynamics. NeuroImage. (2010) 49:3039–46. doi: 10.1016/j.neuroimage.2009.11.050, PMID: 19945536 PMC2818571

[21] Obrig H Villringer A. Beyond the visible--imaging the human brain with light. J Cereb Blood Flow Metab. (2003) 23:1–18. doi: 10.1097/01.WCB.0000043472.45775.29, PMID: 12500086

[22] Metzger FG Ehlis AC Haeussinger FB Schneeweiss P Hudak J Fallgatter AJ. Functional brain imaging of walking while talking - an fNIRS study. Neuroscience. (2017) 343:85–93. doi: 10.1016/j.neuroscience.2016.11.032, PMID: 27915210

[23] Scarapicchia V Brown C Mayo C Gawryluk JR. Functional magnetic resonance imaging and functional near-infrared spectroscopy: insights from combined recording studies. Front Hum Neurosci. (2017) 11:419. doi: 10.3389/fnhum.2017.00419, PMID: 28867998 PMC5563305

[24] Hoshi Y. Functional near-infrared spectroscopy: potential and limitations in neuroimaging studies. Int Rev Neurobiol. (2005) 66:237–66. doi: 10.1016/S0074-7742(05)66008-4, PMID: 16387206

[25] Quaresima V Ferrari M. Functional near-infrared spectroscopy (fNIRS) for assessing cerebral cortex function during human behavior in natural/social situations: a concise review. Organ Res Methods. (2019) 22:46–68. doi: 10.1177/1094428116658959

[26] Kohl SH Mehler DMA Lührs M Thibault RT Konrad K Sorger B. The potential of functional near-infrared spectroscopy-based neurofeedback-a systematic review and recommendations for best practice. Front Neurosci. (2020) 14:594. doi: 10.3389/fnins.2020.00594, PMID: 32848528 PMC7396619

[27] Kubota M Zouridakis G. Differentiation of task complexity in long-term memory retrieval using multifractal detrended fluctuation analysis of fNIRS recordings. Exp Brain Res. (2022) 240:1701–11. doi: 10.1007/s00221-022-06365-z, PMID: 35461464

[28] Li M Yang Y Zhang Y Gao Y Jing R Dang Y. Detecting residual awareness in patients with prolonged disorders of consciousness: an fNIRS study. Front Neurol. (2021) 12:618055. doi: 10.3389/fneur.2021.618055, PMID: 34393964 PMC8355369

[29] Üçeyler N Zeller J Kewenig S Kittel-Schneider S Fallgatter AJ Sommer C. Increased cortical activation upon painful stimulation in fibromyalgia syndrome. BMC Neurol. (2015) 15:210. doi: 10.1186/s12883-015-0472-4, PMID: 26486985 PMC4618366

[30] Dai Y Huang F Zhu Y. Clinical efficacy of motor imagery therapy based on fNIRs technology in rehabilitation of upper limb function after acute cerebral infarction. Pak J Med Sci. (2022) 38:1980–5. doi: 10.12669/pjms.38.7.5344, PMID: 36246721 PMC9532632

[31] He Y Wang N Liu D Peng H Yin S Wang X. Assessment of residual awareness in patients with disorders of consciousness using functional near-infrared spectroscopy-based connectivity: a pilot study. Neurophotonics. (2024) 11:045013. doi: 10.1117/1.NPh.11.4.045013, PMID: 39668847 PMC11635295

[32] Lu J Wu J Shu Z Zhang X Li H Liang S. Brain temporal-spectral functional variability reveals neural improvements of DBS treatment for disorders of consciousness. IEEE Trans Neural Syst Rehabil Eng. (2024) 32:923–33. doi: 10.1109/TNSRE.2024.3368434, PMID: 38386574

[33] Zhang Y Yang Y Si J Xia X He J Jiang T. Influence of inter-stimulus interval of spinal cord stimulation in patients with disorders of consciousness: a preliminary functional near-infrared spectroscopy study. Neuroimage Clin. (2018) 17:1–9. doi: 10.1016/j.nicl.2017.09.017, PMID: 29619317 PMC5883216

[34] Sato T Nambu I Takeda K Aihara T Yamashita O Isogaya Y. Reduction of global interference of scalp-hemodynamics in functional near-infrared spectroscopy using short distance probes. NeuroImage. (2016) 141:120–32. doi: 10.1016/j.neuroimage.2016.06.054, PMID: 27374729

[35] Delpy DT Cope M Pvd Z Arridge S Wray S Wyatt J. Estimation of optical pathlength through tissue from direct time of flight measurement. Phys Med Biol. (1988) 33:1433–42. doi: 10.1088/0031-9155/33/12/008, PMID: 3237772

[36] Kempny AM James L Yelden K Duport S Farmer S Playford ED. Functional near infrared spectroscopy as a probe of brain function in people with prolonged disorders of consciousness. Neuroimage Clin. (2016) 12:312–9. doi: 10.1016/j.nicl.2016.07.013, PMID: 27547728 PMC4983150

[37] Ferrari M Quaresima V. Near infrared brain and muscle oximetry: from the discovery to current applications. J Near Infrared Spectrosc. (2012) 20:1–14. doi: 10.1255/jnirs.973

[38] Fantini S Sassaroli A. Near-infrared optical mammography for breast cancer detection with intrinsic contrast. Ann Biomed Eng. (2012) 40:398–407. doi: 10.1007/s10439-011-0404-4, PMID: 21971964 PMC3678374

[39] Doulgerakis M Eggebrecht AT Dehghani H. High-density functional diffuse optical tomography based on frequency-domain measurements improves image quality and spatial resolution. Neurophotonics. (2019) 6:1. doi: 10.1117/1.NPh.6.3.035007, PMID: 31482102 PMC6702521

[40] Toronov VY Zhang X Webb AG. A spatial and temporal comparison of hemodynamic signals measured using optical and functional magnetic resonance imaging during activation in the human primary visual cortex. NeuroImage. (2007) 34:1136–48. doi: 10.1016/j.neuroimage.2006.08.048, PMID: 17134913 PMC2752293

[41] Jurcak V Tsuzuki D Dan I. 10/20, 10/10, and 10/5 systems revisited: their validity as relative head-surface-based positioning systems. NeuroImage. (2007) 34:1600–11. doi: 10.1016/j.neuroimage.2006.09.024, PMID: 17207640

[42] Pinti P Tachtsidis I Hamilton A Hirsch J Aichelburg C Gilbert S. The present and future use of functional near-infrared spectroscopy (fNIRS) for cognitive neuroscience. Ann N Y Acad Sci. (2020) 1464:5–29. doi: 10.1111/nyas.13948, PMID: 30085354 PMC6367070

[43] Ferrari M Quaresima V. A brief review on the history of human functional near-infrared spectroscopy (fNIRS) development and fields of application. NeuroImage. (2012) 63:921–35. doi: 10.1016/j.neuroimage.2012.03.049, PMID: 22510258

[44] Cui X Bray S Bryant DM Glover GH Reiss AL. A quantitative comparison of NIRS and fMRI across multiple cognitive tasks. NeuroImage. (2011) 54:2808–21. doi: 10.1016/j.neuroimage.2010.10.069, PMID: 21047559 PMC3021967

[45] Zhang X Noah JA Hirsch J. Separation of the global and local components in functional near-infrared spectroscopy signals using principal component spatial filtering. Neurophotonics. (2016) 3:015004. doi: 10.1117/1.NPh.3.1.015004, PMID: 26866047 PMC4742567

[46] Yucel MA Luhmann AV Scholkmann F Gervain J Dan I Ayaz H. Best practices for fNIRS publications. Neurophotonics. (2021) 8:012101. doi: 10.1117/1.NPh.8.1.012101, PMID: 33442557 PMC7793571

[47] Stefanovska A Bracic M Kvernmo HD. Wavelet analysis of oscillations in the peripheral blood circulation measured by laser Doppler technique. IEEE Trans Biomed Eng. (1999) 46:1230–9. doi: 10.1109/10.790500, PMID: 10513128

[48] Scholkmann F Gerber U Wolf M Wolf U. End-tidal CO2: an important parameter for a correct interpretation in functional brain studies using speech tasks. NeuroImage. (2013) 66:71–9. doi: 10.1016/j.neuroimage.2012.10.025, PMID: 23099101

[49] Abdelnour AF Huppert T. Real-time imaging of human brain function by near-infrared spectroscopy using an adaptive general linear model. NeuroImage. (2009) 46:133–43. doi: 10.1016/j.neuroimage.2009.01.033, PMID: 19457389 PMC2758631

[50] Yücel MA Selb J Aasted CM Lin PY Borsook D Becerra L. Mayer waves reduce the accuracy of estimated hemodynamic response functions in functional near-infrared spectroscopy. Biomed Opt Express. (2016) 7:3078–88. doi: 10.1364/BOE.7.003078, PMID: 27570699 PMC4986815

[51] Sabrina B Robert JC. How short is short? Optimum source–detector distance for short-separation channels in functional near-infrared spectroscopy. Neurophotonics. (2015) 2:025005. doi: 10.1117/1.NPh.2.2.025005, PMID: 26158009 PMC4478880

[52] Zhou X Sobczak G McKay CM Litovsky RY. Comparing fNIRS signal qualities between approaches with and without short channels. PLoS One. (2020) 15:e0244186. doi: 10.1371/journal.pone.0244186, PMID: 33362260 PMC7757903

[53] Noah JA Xian ZZ Swethasri D DiCocco C Suzuki T Aslin RN. Comparison of short-channel separation and spatial domain filtering for removal of non-neural components in functional near-infrared spectroscopy signals. Neurophotonics. (2021) 8:015004. doi: 10.1117/1.NPh.8.1.015004, PMID: 33598505 PMC7881368

[54] Miguel HO Condy EE Nguyen T Zeytinoglu S Blick E Bress K. Cerebral hemodynamic response during a live action-observation and action-execution task: a fNIRS study. PLoS One. (2021) 16:e0253788. doi: 10.1371/journal.pone.0253788, PMID: 34388157 PMC8362964

[55] Cooper RJ Selb J Gagnon L Phillip D Schytz HW Iversen HK. A systematic comparison of motion artifact correction techniques for functional near-infrared spectroscopy. Front Neurosci. (2012) 6:147. doi: 10.3389/fnins.2012.00147, PMID: 23087603 PMC3468891

[56] Liu G Huo E Liu H Jia G Zhi Y Dong Q. Development and emergence of functional network asymmetry in 3- to 9-month-old infants. Cortex. (2022) 154:390–404. doi: 10.1016/j.cortex.2022.06.009, PMID: 35930891

[57] Zhao Y Luo H Chen J Loureiro R Yang S Zhao H. Learning based motion artifacts processing in fNIRS: a mini review. Front Neurosci. (2023) 17:1280590. doi: 10.3389/fnins.2023.1280590, PMID: 38033535 PMC10683641

[58] Huang R Hong KS Yang D Huang G. Motion artifacts removal and evaluation techniques for functional near-infrared spectroscopy signals: a review. Front Neurosci. (2022) 16:878750. doi: 10.3389/fnins.2022.878750, PMID: 36263362 PMC9576156

[59] Perrey S. Non-invasive NIR spectroscopy of human brain function during exercise. Methods. (2008) 45:289–99. doi: 10.1016/j.ymeth.2008.04.005, PMID: 18539160

[60] Huppert TJ Diamond SG Franceschini MA Boas DA. HomER: a review of time-series analysis methods for near-infrared spectroscopy of the brain. Appl Opt. (2009) 48:D280–98. doi: 10.1364/AO.48.00D280, PMID: 19340120 PMC2761652

[61] Zhang H Yang J Ni J De Dreu CKW Ma Y. Leader-follower behavioural coordination and neural synchronization during intergroup conflict. Nat Hum Behav. (2023) 7:2169–81. doi: 10.1038/s41562-023-01663-0, PMID: 37500783

[62] Mark JA Curtin A Kraft AE Ziegler MD Ayaz H. Mental workload assessment by monitoring brain, heart, and eye with six biomedical modalities during six cognitive tasks. Front Neuroergon. (2024) 5:1345507. doi: 10.3389/fnrgo.2024.1345507, PMID: 38533517 PMC10963413

[63] Karunakaran KD Peng K Berry D Green S Labadie R Kussman B. NIRS measures in pain and analgesia: fundamentals, features, and function. Neurosci Biobehav Rev. (2021) 120:335–53. doi: 10.1016/j.neubiorev.2020.10.023, PMID: 33159918

[64] Khaksari K Chen WL Chanvanichtrakool M Taylor A Kotla R Gropman AL. Applications of near-infrared spectroscopy in epilepsy, with a focus on mitochondrial disorders. Neurotherapeutics. (2024) 21:e00323. doi: 10.1016/j.neurot.2024.e00323, PMID: 38244258 PMC10903079

[65] Zhao YN Han PP Zhang XY Bi X. Applications of functional near-infrared spectroscopy (fNIRS) neuroimaging during rehabilitation following stroke: a review. Med Sci Monit. (2024) 30:e943785. doi: 10.12659/MSM.943785, PMID: 38879751 PMC11188690

[66] Bullmore E Sporns O. Complex brain networks: graph theoretical analysis of structural and functional systems. Nat Rev Neurosci. (2009) 10:186–98. doi: 10.1038/nrn2575, PMID: 19190637

[67] Avena-Koenigsberger A Misic B Sporns O. Communication dynamics in complex brain networks. Nat Rev Neurosci. (2017) 19:17–33. doi: 10.1038/nrn.2017.149, PMID: 29238085

[68] Fingelkurts AA Fingelkurts AA Kähkönen S. Functional connectivity in the brain--is it an elusive concept? Neurosci Biobehav Rev. (2005) 28:827–36. doi: 10.1016/j.neubiorev.2004.10.009, PMID: 15642624

[69] Bullmore E Sporns O. The economy of brain network organization. Nat Rev Neurosci. (2012) 13:336–49. doi: 10.1038/nrn3214, PMID: 22498897

[70] Yang Y Dai Y He Q Wang S Chen X Geng X. Altered brain functional connectivity in vegetative state and minimally conscious state. Front Aging Neurosci. (2023) 15:1213904. doi: 10.3389/fnagi.2023.1213904, PMID: 37469954 PMC10352323

[71] Sinitsyn DO Legostaeva LA Kremneva EI Morozova SN Poydasheva AG Mochalova EG. Degrees of functional connectome abnormality in disorders of consciousness. Hum Brain Mapp. (2018) 39:2929–40. doi: 10.1002/hbm.24050, PMID: 29575425 PMC6866294

[72] Mashour GA Roelfsema P Changeux JP Dehaene S. Conscious processing and the global neuronal workspace hypothesis. Neuron. (2020) 105:776–98. doi: 10.1016/j.neuron.2020.01.026, PMID: 32135090 PMC8770991

[73] Demertzi A Antonopoulos G Heine L Voss HU Crone JS de Los Angeles C. Intrinsic functional connectivity differentiates minimally conscious from unresponsive patients. Brain. (2015) 138:2619–31. doi: 10.1093/brain/awv169, PMID: 26117367

[74] Chen H Miao G Wang S Zheng J Zhang X Lin J. Disturbed functional connectivity and topological properties of the frontal lobe in minimally conscious state based on resting-state fNIRS. Front Neurosci. (2023) 17:1118395. doi: 10.3389/fnins.2023.1118395, PMID: 36845431 PMC9950516

[75] Naccache L. Minimally conscious state or cortically mediated state? Brain. (2018) 141:949–60. doi: 10.1093/brain/awx324, PMID: 29206895 PMC5888986

[76] Demertzi A Sitt JD Sarasso S Pinxten W. Measuring states of pathological (un)consciousness: research dimensions, clinical applications, and ethics. Neurosci Conscious. (2017) 2017:nix010. doi: 10.1093/nc/nix010, PMID: 30042843 PMC6007135

[77] Noirhomme Q Brecheisen R Lesenfants D Antonopoulos G Laureys S. "look at my classifier's result": disentangling unresponsive from (minimally) conscious patients. NeuroImage. (2017) 145:288–303. doi: 10.1016/j.neuroimage.2015.12.006, PMID: 26690804

[78] Zhang H Duan L Zhang YJ Lu CM Liu H Zhu CZ. Test-retest assessment of independent component analysis-derived resting-state functional connectivity based on functional near-infrared spectroscopy. NeuroImage. (2011) 55:607–15. doi: 10.1016/j.neuroimage.2010.12.007, PMID: 21146616

[79] Lu CM Zhang YJ Biswal BB Zang YF Peng DL Zhu CZ. Use of fNIRS to assess resting state functional connectivity. J Neurosci Methods. (2010) 186:242–9. doi: 10.1016/j.jneumeth.2009.11.010, PMID: 19931310

[80] Liu Y Kang XG Chen BB Song CG Liu Y Hao JM. Detecting residual brain networks in disorders of consciousness: a resting-state fNIRS study. Brain Res. (2023) 1798:148162. doi: 10.1016/j.brainres.2022.148162, PMID: 36375509

[81] Urquhart EL Wang X Liu H Fadel PJ Alexandrakis G. Differences in net information flow and dynamic connectivity metrics between physically active and inactive subjects measured by functional near-infrared spectroscopy (fNIRS) during a fatiguing handgrip task. Front Neurosci. (2020) 14:167. doi: 10.3389/fnins.2020.00167, PMID: 32210748 PMC7076120

[82] Guillot A Collet C Nguyen VA Malouin F Richards C Doyon J. Brain activity during visual versus kinesthetic imagery: an fMRI study. Hum Brain Mapp. (2009) 30:2157–72. doi: 10.1002/hbm.20658, PMID: 18819106 PMC6870928

[83] Si J Yang Y Xu L Xu T Liu H Zhang Y. Evaluation of residual cognition in patients with disorders of consciousness based on functional near-infrared spectroscopy. Neurophotonics. (2023) 10:025003. doi: 10.1117/1.NPh.10.2.025003, PMID: 37064779 PMC10091901

[84] di H Yu SM Weng XC Laureys S Yu D Li J. Cerebral response to patient's own name in the vegetative and minimally conscious states. Neurology. (2007) 68:895–9. doi: 10.1212/01.wnl.0000258544.79024.d0, PMID: 17372124

[85] GBD 2016 Epilepsy Collaborators. Global, regional, and national burden of epilepsy, 1990-2016: a systematic analysis for the global burden of disease study 2016. Lancet Neurol. (2019) 18:357–75. doi: 10.1016/S1474-4422(18)30454-X, PMID: 30773428 PMC6416168

[86] Lu J Xu X Huang Y Li T Ma C Xu G. Prevalence of depressive disorders and treatment in China: a cross-sectional epidemiological study. Lancet Psychiatry. (2021) 8:981–90. doi: 10.1016/S2215-0366(21)00251-0, PMID: 34559991

[87] Zhu Z Hu X Mao Y. Spinal cord electrical stimulation for severe disturbance of consciousness after traumatic brain injury: a case report. Heliyon. (2024) 10:e34913. doi: 10.1016/j.heliyon.2024.e34913, PMID: 39144968 PMC11320212

[88] Lee DJ Lozano CS Dallapiazza RF Lozano AM. Current and future directions of deep brain stimulation for neurological and psychiatric disorders. J Neurosurg. (2019) 131:333–42. doi: 10.3171/2019.4.JNS181761, PMID: 31370011

[89] Tasserie J Uhrig L Sitt JD Manasova D Dupont M Dehaene S. Deep brain stimulation of the thalamus restores signatures of consciousness in a nonhuman primate model. Sci Adv. (2022) 8:eabl5547. doi: 10.1126/sciadv.abl5547, PMID: 35302854 PMC8932660

[90] Hassler R Ore GD Bricolo A Dieckmann G Dolce G. EEG and clinical arousal induced by bilateral long-term stimulation of pallidal systems in traumatic vigil coma. Electroencephalogr Clin Neurophysiol. (1969) 27:689–90. doi: 10.1016/0013-4694(69)91313-3, PMID: 4187363

[91] Shu Z Wu J Li H Liu J Lu J Lin J. fNIRS-based functional connectivity signifies recovery in patients with disorders of consciousness after DBS treatment. Clin Neurophysiol. (2023) 147:60–8. doi: 10.1016/j.clinph.2022.12.011, PMID: 36702043

[92] Si J Dang Y Zhang Y Li Y Zhang W Yang Y. Spinal cord stimulation frequency influences the hemodynamic response in patients with disorders of consciousness. Neurosci Bull. (2018) 34:659–67. doi: 10.1007/s12264-018-0252-4, PMID: 29995275 PMC6060214

[93] Naito M Michioka Y Ozawa K Ito Y Kiguchi M Kanazawa T. A communication means for totally locked-in ALS patients based on changes in cerebral blood volume measured with near-infrared light. IEICE Trans Inf Syst. (2007) E90-D:1028–37. doi: 10.1093/ietisy/e90-d.7.1028, PMID: 39816196

[94] Vorreuther A Bastian L Benitez Andonegui A Evenblij D Riecke L Lührs M. It takes two (seconds): decreasing encoding time for two-choice functional near-infrared spectroscopy brain-computer interface communication. Neurophotonics. (2023) 10:045005. doi: 10.1117/1.NPh.10.4.045005, PMID: 37928600 PMC10620514

[95] Fernández-Espejo D Rossit S Owen AM. A Thalamocortical mechanism for the absence of overt motor behavior in covertly aware patients. JAMA Neurol. (2015) 72:1442–50. doi: 10.1001/jamaneurol.2015.2614, PMID: 26501399

[96] Coleman MR Rodd JM Davis MH Johnsrude IS Menon DK Pickard JD. Do vegetative patients retain aspects of language comprehension? Evidence from fMRI. Brain. (2007) 130:2494–507. doi: 10.1093/brain/awm170, PMID: 17827174

[97] Coleman MR Davis MH Rodd JM Robson T Ali A Owen AM. Towards the routine use of brain imaging to aid the clinical diagnosis of disorders of consciousness. Brain. (2009) 132:2541–52. doi: 10.1093/brain/awp183, PMID: 19710182

[98] Ma Y Yu Y Gao W Hong Y Shen X. Cerebral hemodynamic changes during unaffected handgrip exercises in stroke patients: an fNIRS study. Brain Sci. (2023) 13:141. doi: 10.3390/brainsci13010141, PMID: 36672122 PMC9857146

[99] Wang Z Yang L Zhou Y Chen L Gu B Liu S. Incorporating EEG and fNIRS patterns to evaluate cortical excitability and MI-BCI performance during motor training. IEEE Trans Neural Syst Rehabil Eng. (2023) 31:2872–82. doi: 10.1109/TNSRE.2023.3281855, PMID: 37262121

[100] Naseer N Hong KS. fNIRS-based brain-computer interfaces: a review. Front Hum Neurosci. (2015) 9:3. doi: 10.3389/fnhum.2015.00003, PMID: 25674060 PMC4309034

[101] Artemenko C Soltanlou M Ehlis AC Nuerk HC Dresler T. The neural correlates of mental arithmetic in adolescents: a longitudinal fNIRS study. Behav Brain Funct. (2018) 14:5. doi: 10.1186/s12993-018-0137-8, PMID: 29524965 PMC5845230

[102] Zhang Y Liu D Li T Zhang P Li Z Gao F. CGAN-rIRN: a data-augmented deep learning approach to accurate classification of mental tasks for a fNIRS-based brain-computer interface. Biomed Opt Express. (2023) 14:2934–54. doi: 10.1364/BOE.489179, PMID: 37342712 PMC10278643

[103] Kwon J Im CH. Subject-independent functional near-infrared spectroscopy-based brain-computer interfaces based on convolutional neural networks. Front Hum Neurosci. (2021) 15:646915. doi: 10.3389/fnhum.2021.646915, PMID: 33776674 PMC7994252

[104] Nazeer H Naseer N Mehboob A Khan MJ Khan RA Khan US. Enhancing classification performance of fNIRS-BCI by identifying cortically active channels using the z-score method. Sensors (Basel). (2020) 20:6995. doi: 10.3390/s20236995, PMID: 33297516 PMC7730208

[105] Guger C Spataro R Allison BZ Heilinger A Ortner R Cho W. Complete locked-in and locked-in patients: command following assessment and communication with Vibro-tactile P300 and motor imagery brain-computer Interface tools. Front Neurosci. (2017) 11:251. doi: 10.3389/fnins.2017.00251, PMID: 28529473 PMC5418541

[106] Wang F He Y Qu J Xie Q Lin Q Ni X. Enhancing clinical communication assessments using an audiovisual BCI for patients with disorders of consciousness. J Neural Eng. (2017) 14:046024. doi: 10.1088/1741-2552/aa6c31, PMID: 28393761

[107] Rahman MA Siddik AB Ghosh TK Khanam F Ahmad M. A narrative review on clinical applications of fNIRS. J Digit Imaging. (2020) 33:1167–84. doi: 10.1007/s10278-020-00387-1, PMID: 32989620 PMC7573058

[108] Irani F Platek SM Bunce S Ruocco AC Chute D. Functional near infrared spectroscopy (fNIRS): an emerging neuroimaging technology with important applications for the study of brain disorders. Clin Neuropsychol. (2007) 21:9–37. doi: 10.1080/13854040600910018, PMID: 17366276

[109] Abdalmalak A Milej D Cohen DJ Anazodo U Ssali T Diop M. Using fMRI to investigate the potential cause of inverse oxygenation reported in fNIRS studies of motor imagery. Neurosci Lett. (2020) 714:134607. doi: 10.1016/j.neulet.2019.134607, PMID: 31693928

[110] Yücel MA Selb J Boas DA Cash SS Cooper RJ. Reducing motion artifacts for long-term clinical NIRS monitoring using collodion-fixed prism-based optical fibers. NeuroImage. (2014) 85:192–201. doi: 10.1016/j.neuroimage.2013.06.054, PMID: 23796546 PMC3849205

[111] Kazazian K Norton L Laforge G Abdalmalak A Gofton TE Debicki D. Improving diagnosis and prognosis in acute severe brain injury: a multimodal imaging protocol. Front Neurol. (2021) 12:757219. doi: 10.3389/fneur.2021.757219, PMID: 34938260 PMC8685572

[112] Rupawala M Dehghani H Lucas SJE Tino P Cruse D. Shining a light on awareness: a review of functional near-infrared spectroscopy for prolonged disorders of consciousness. Front Neurol. (2018) 9:350. doi: 10.3389/fneur.2018.00350, PMID: 29872420 PMC5972220

[113] Othman MH Bhattacharya M Møller K Kjeldsen S Grand J Kjaergaard J. Resting-state NIRS-EEG in unresponsive patients with acute brain injury: a proof-of-concept study. Neurocrit Care. (2021) 34:31–44. doi: 10.1007/s12028-020-00971-x, PMID: 32333214

[114] Liu N Cui X Bryant DM Glover GH Reiss AL. Inferring deep-brain activity from cortical activity using functional near-infrared spectroscopy. Biomed Opt Express. (2015) 6:1074–89. doi: 10.1364/BOE.6.001074, PMID: 25798327 PMC4361422

[115] Cui X Bryant DM Reiss AL. NIRS-based hyperscanning reveals increased interpersonal coherence in superior frontal cortex during cooperation. NeuroImage. (2012) 59:2430–7. doi: 10.1016/j.neuroimage.2011.09.003, PMID: 21933717 PMC3254802

[116] Hirsch J Zhang X Noah JA Ono Y. Frontal temporal and parietal systems synchronize within and across brains during live eye-to-eye contact. NeuroImage. (2017) 157:314–30. doi: 10.1016/j.neuroimage.2017.06.018, PMID: 28619652 PMC5863547

[117] Moriya M Sakatani K. Relation between asymmetry of prefrontal activity and autonomic nervous system in post-stroke patients with a disorder of consciousness. Adv Exp Med Biol. (2018) 1:53–8. doi: 10.1007/978-3-319-91287-5_9 30178323

[118] Bicciato G Narula G Brandi G Eisele A Schulthess S Friedl S. Functional NIRS to detect covert consciousness in neurocritical patients. Clin Neurophysiol. (2022) 144:72–82. doi: 10.1016/j.clinph.2022.10.002 36306692

[119] Luo Y Wang L Yang Y Jiang X Zheng K Xi Y. Exploration of resting-state brain functional connectivity as preclinical markers for arousal prediction in prolonged disorders of consciousness: A pilot study based on functional near-infrared spectroscopy. Brain Behav. (2024) 14:e70002. doi: 10.1002/brb3.70002 39183500 PMC11345494

[120] Xin W Liu Z Shao Y Peng Y Liu H Wang M. Effects of acupuncture on cortical activation in patients with disorders of consciousness: a functional near-infrared spectroscopy study. Evid Based Complement Alternat Med. (2022) 2022:5711961. doi: 10.1155/2022/5711961 35958938 PMC9363174

[121] Liu Y Sun N Xiong J Zhou Y Ye X Jiang H. Modulation of cerebral cortex activity by acupuncture in patients with prolonged disorder of consciousness: An fNIRS study. Front Neurosci. (2022) 16:1043133. doi: 10.3389/fnins.2022.1043133 36523434 PMC9744766

[122] Straudi S Antonioni A Baroni A Bonsangue V Lavezzi S Koch G. Anti-inflammatory and cortical responses after transcranial direct current stimulation in disorders of consciousness: an exploratory study. J Clin Med. (2023) 13:108. doi: 10.3390/jcm13010108 38202115 PMC10779892

[123] Shu Z Wu J Lu J Li H Liu J Lin J. Effective DBS treatment improves neural information transmission of patients with disorders of consciousness: an fNIRS study. Physiol Meas. (2023) 44. doi: 10.1088/1361-6579/ad14ab 38086065

[124] Yin J Xu G Xie H Liu Y Dou Z Shao B. Effects of different frequencies music on cortical responses and functional connectivity in patients with minimal conscious state. J Biophotonics. (2024) 17:e202300427. doi: 10.1002/jbio.202300427 38303080

[125] Lu H Jiang J Si J Wang Y Huang F. A functional near-infrared spectroscopy study on hemodynamic changes of patients with prolonged disorders of consciousness responding to different auditory stimuli. BMC Neurol. (2023) 23:242. doi: 10.1186/s12883-023-03292-6 37353754 PMC10288743

[126] Abdalmalak A Milej D Norton L Debicki DB Gofton T Diop M. Single-session communication with a locked-in patient by functional near-infrared spectroscopy. Neurophotonics. (2017) 4:040501. doi: 10.1117/1.NPh.4.4.040501 29296627 PMC5741990

[127] Kurz EM Wood G Kober SE Schippinger W Pichler G Müller-Putz G. Towards using fNIRS recordings of mental arithmetic for the detection of residual cognitive activity in patients with disorders of consciousness (DOC). Brain Cogn. (2018) 125:78–87. doi: 10.1016/j.bandc.2018.06.002 29909026

